# Neutrophil extracellular traps in CSF and serum of dogs with steroid-responsive meningitis-arteritis

**DOI:** 10.1371/journal.pone.0295268

**Published:** 2024-01-19

**Authors:** Jan Christian Wohlsein, Marita Meurer, Matthias Mörgelin, Jasmin Nicole Nessler, Thomas Flegel, Henning Christian Schenk, Konrad Jurina, Kai Rentmeister, Andrea Fischer, Thomas Gödde, Wolfgang Baumgärtner, Maren von Köckritz-Blickwede, Andrea Tipold

**Affiliations:** 1 Department of Small Animal Medicine and Surgery, University of Veterinary Medicine Hannover, Foundation, Hannover, Germany; 2 Department of Biochemistry, University of Veterinary Medicine Hannover, Foundation, Hannover, Germany; 3 Research Centre for Emerging Infections and Zoonoses, University of Veterinary Medicine Hannover, Foundation, Hannover, Germany; 4 Colzyx AB, Medicon Village, Lund, Sweden; 5 Department for Small Animals, Faculty of Veterinary Medicine, Leipzig University, Leipzig, Germany; 6 Tierklinik Lüneburg, Lüneburg, Germany; 7 AniCura Tierklinik Haar, Haar, Germany; 8 Tieraerztliche Praxis für Neurologie, Small Animal Practice, Dettelbach, Germany; 9 Clinic of Small Animal Medicine, Centre for Clinical Veterinary Medicine, Ludwig-Maximilians-University Munich, Munich, Germany; 10 Small Animal Clinic Piding, Piding, Germany; 11 Department of Pathology, University of Veterinary Medicine Hannover, Foundation, Hannover, Germany; Khalifa University of Science and Technology, UNITED ARAB EMIRATES

## Abstract

In steroid-responsive meningitis-arteritis (SRMA), inflammatory dysregulation is driven by neutrophilic granulocytes resulting in purulent leptomeningitis. Neutrophils can generate neutrophil extracellular traps (NET). Uncontrolled NET-formation or impaired NET-clearance evidently cause tissue and organ damage resulting in immune-mediated diseases. The aim of the study was to verify that NET-formation is detectable in *ex vivo* samples of acute diseased dogs with SRMA by visualizing and measuring NET-markers in serum and cerebrospinal fluid (CSF) samples. CSF-samples of dogs with acute SRMA (n = 5) and in remission (n = 4) were examined using immunofluorescence (IF)-staining of DNA-histone-1-complexes, myeloperoxidase and citrullinated Histone H3 (H3Cit). Immunogold-labeling of H3Cit and neutrophil elastase followed by transmission electron microscopy (TEM) were used to determine ultrastructural NET-formation in the CSF of one exemplary dog. H3Cit-levels and DNase-activity were measured in CSF and serum samples using an H3Cit-ELISA and a DNase-activity-assay, respectively in patients with the following diseases: acute SRMA (n = 34), SRMA in remission (n = 4), bacterial encephalitis (n = 3), meningioma with neutrophilic inflammation (n = 4), healthy dogs (n = 6). NET-formation was detectable with IF-staining in n = 3/5 CSF samples of dogs with acute SRMA but were not detectable during remission. Vesicular NET-formation was detectable in one exemplary dog using TEM. DNase-activity was significantly reduced in dogs suffering from acute SRMA compared to healthy control group (*p* < 0.0001). There were no statistical differences of H3Cit levels in CSF or serum samples of acute diseased dogs compared to dogs under treatment, dogs suffering from meningioma or bacterial encephalitis or the healthy control group. Our findings demonstrate that NET-formation and insufficient NET-clearance possibly drive the immunologic dysregulation and complement the pathogenesis of SRMA. The detection of NETs in SRMA offers many possibilities to explore the aetiopathogenetic influence of this defence mechanism of the innate immune system in infectious and non-infectious canine neuropathies.

## Introduction/Background

Steroid responsive meningitis-arteritis (SRMA) is a systemic, immune-mediated disorder in dogs characterized by severe inflammatory and degenerative alterations of leptomeningeal vessels and neutrophilic meningitis especially in the cervical meninges [1, 2]. SRMA represents the most common meningitis and reason of pyrexia as well as cervical hyperesthesia in young dogs [3–6]. The definitive aetiology and pathogenesis of SRMA remains elusive even though multiple potential aetiologies or disease triggers have been investigated in the past [7, 8]. Effective immunosuppressive therapy leads to the assumption of an immune-mediated disorder [1, 8]. Mainly juvenile-adult, medium to large sized dog breeds are preferentially affected [1]. Overrepresented breeds are Bernese Mountain dogs, Beagles [8, 9], Boxer, Weimaraners [1], Nova Scotia Duck Tolling Retrievers [10], and Petit Basset Griffon Vendeéns [11] implicating the existence of a genetic predisposition.

Clinical signs of SRMA are episodic and recurrent. The disorder can be divided in two forms: the acute, typical or the chronic, atypical respective protracted form [1]. On the one hand, dogs suffer from signs of systemic inflammation like pyrexia and lethargy in the acute form and on the other hand of severe cervical leptomeningitis with neck pain resulting in hunched posture, stiff gait, and reluctance to move [9, 12]. Protracted cases of the atypical form occur due to relapses and insufficient control of the immunologic response by immunosuppressive treatment and may appear with cranial nerve deficits, ataxia, and paresis [9].

The combination of signalment, clinical signs, and laboratory alterations of cerebrospinal fluid (CSF), and hematology analysis in association with a fast improvement of clinical symptoms after administration of glucocorticosteroids are indicative for the diagnosis SRMA [1, 5, 13]. A supportive, but not pathognomonic hematologic finding is a moderate to severe neutrophilic leucocytosis with left shift [8, 9]. Elevated serum levels of acute phase proteins for example C-reactive protein (CRP) can be detected and support the monitoring of therapy success and remission efficiently [14, 15]. The CSF examination represents the crucial laboratory test for SRMA. Macroscopically the CSF may appear turbid in case of severe pleocytosis and xanthochromic because of hemorrhage [2]. Increased protein concentrations and total nucleated cell count of the CSF especially dominated by non-degenerated neutrophils indicate distinctive laboratory alterations of SRMA [9, 16]. Intrathecal and systemic elevated immunoglobulin A (IgA) levels sustain the diagnosis with high specificity (91%) and sensitivity (78%) [17].

Predominant histopathologic findings reveal fibrinoid-necrotizing arteritis of small and medium-sized arteries and multifocal to generalized, purulent leptomeningitis most commonly in the meninges of the cervical spinal cord [1, 3, 12, 18, 19]. The characteristic systemic and intrathecal neutrophilia is regulated by several immunologic mechanisms. The detailed trigger of this example of immune compartmentalization remains obscure. Selective upregulation of CD11a-expression on neutrophils [16], production of matrix metalloproteases 2 and 9 (MMP-2/-9) [20], chemotactic triggers of Interleukin (IL)– 8 generated in the central nervous system (CNS) [21], IL-6 produced by macrophages [22], and IL–17 produced by upregulated Th17-cells [23] facilitate the severe neutrophilic crossover of the blood brain barrier and conquest of the subarachnoid space. Besides neutrophils, macrophages, CD4+ cells, plasma cells and lymphocytes are present during the inflammatory process and ensure an orchestrated interaction of the meningeal inflammation and severe arteritis [3, 19, 24–26].

Since the proinflammatory milieu of cytokines e. g. IL-17 is hypothesized to stimulate NET-formation, it may be speculated that NETs also play a role in SRMA [27]. These specific conditions of the unique pathogenesis of SRMA emerging with systemic and characteristic intrathecal neutrophilia display ideal circumstances to investigate a potential formation of neutrophil extracellular traps (NETs) as possible part of the immunologic dysregulation [1, 9]. As sentinel cells of the innate immune system neutrophils serve in the first line of defense to fight infectious diseases [28]. Their multifaceted antimicrobial weapons include degranulation of intracellular stored proteinases and antimicrobial peptides, respiratory burst by generating reactive oxygen species and phagocytosis [28–30]. In 2004, Brinkmann et al. [31] described another extracellular strategy of neutrophils entrapping and killing pathogenic microorganisms in externalized, three-dimensional, web-like organized structures of decondensed chromatin. This chromatin backbone is equipped with histones antimicrobial peptides and granule proteins including myeloperoxidase (MPO), neutrophil elastase (NE) and proteinase 3 (PR3) [31].

NET-formation occurs mainly in three different pathways. Suicidal NETosis is characterized by lyses of the cell membrane and release of NETs as controlled cell death, which phenotypically differs from necrosis and apoptosis [31, 32]. On the other hand, neutrophils are able to retain cellular homeostasis by releasing vesicular NETs–called vital NET-formation [33–35]. A third poorly understood way was previously described that neutrophils are releasing mitochondrial DNA resulting in extracellular NETs similar to nuclear DNA release which was seen for neutrophil and eosinophil granulocytes [36].

In general, NET-formation represents an immunologic mechanism and appears as double-edged sword by beneficially serving as effective antimicrobial equipment of neutrophils [37]. But on the other hand, if unregulated and excessive NET-formation occurs or NETs are inefficiently cleaved [37–40], biologic active proteins and antimicrobial enzymes may cause detrimental effects on host tissues as described in autoinflammatory mediated processes [37, 41–43], variable entities of immune-mediated vasculitis [44–47], and cancer [48]. Furthermore, the effect of several anti-inflammatory drugs, especially glucocorticosteroids, is controversial concerning their influence on NET-formation. Methylprednisolone induces NET-formation and enhances the bactericidal effect on canine neutrophils [49]. On the other hand, NET-formation is inhibited by steroids during bacterial infections [50, 51].

A crucial mechanism of NET-formation is represented by hypercitrullination of histone H3 catalyzed by peptidylarginin-deiminiase 4 (PAD4) [52]. Hypercitrullination of positively charged arginine residues produces uncharged citrulline residues resulting in loss of ionic interactions and resulting in chromatin decondensation [52–54]. H3Cit was previously described as reliable biomarker for NETosis in different conditions [55–58]. Host deoxyribonucleases (DNases) are essential for ensuring the balance between continuous NET-formation, recycling of NET-structures and their degradation to prohibit the accumulation of NETs and their toxic components [59, 60].

Detailed investigation on NET-formation and NET-metabolism may supplement the neutrophil dominated pathogenesis of a suspected immune-mediated disease such as SRMA which serves as animal model for neutrophil meningitis [23, 26] as well as immune-mediated vasculitis like Kawasaki-disease in children [61, 62]. The aim of the current study was to investigate the appearance of NETs respectively NET-markers in CSF and serum samples of dogs suffering from acute SRMA leading to a better understanding of this disorder and enable possible new therapeutic strategies. To clarify whether NET-formation is present we focused on four different methods in terms of NET-visualization in CSF-samples via immunofluorescence and transmission electron microscopy, as well as measurement of H3Cit-levels and determination of DNase activity in serum and CSF samples.

## Material and methods

### Sample collection, animals, data collection

The study was divided up in a retrospective and prospective approach to prove the occurrence of NET-formation in dogs with acute SRMA. Prospectively NET-formation was investigated in fresh CSF samples from acute diseased dogs by two different methods of visualization. Retrospectively direct (H3Cit) and indirect NET-markers (DNase activity) were measured in archived serum and CSF samples using an H3Cit-ELISA and DNase activity assay.

Fresh CSF samples were collected at the Department for Small Animal Medicine and Surgery of the University of Veterinary Medicine, Hannover, Foundation, Germany, for immunofluorescence (IF) and transmission electron microscopy (TEM). SRMA was diagnosed in dogs with fever >39.0°C, cervical hyperesthesia, blood profiles with leucocytosis (6 x 10^3^/μL to 12 x 10^3^/μL) and neutrophilia (60 to 75%), as well as CSF examination. Increased neutrophilic cell count (>5 leukocytes/μL and >25% neutrophils), increased CSF protein (>25 mg/dL) [1, 5, 7, 13] and elevated levels of IgA in serum (reference range: 10.9–100.1 μg/mL) [17] and CSF (reference range: <0.2 μg/mL) [17] are diagnostic criteria for SRMA after exclusion of an infectious aetiology which could explain the clinical signs of individual dogs. CSF was taken suboccipitally with a 22 Gauge spinal needle of the subarachnoid space in general anesthesia. These CSF samples for immunofluorescence staining and transmission electron microscopy were immediately processed. Only residual samples of routine diagnostic work up were analyzed so that no extra samples were taken for this study.

The retrospective approach of the study analyzed archived serum and CSF samples using an H3Cit ELISA and DNase I activity assay. These samples were collected at the archive of the Department of Small Animal Medicine and Surgery of the University of Veterinary Medicine Hannover, Foundation. These client-owned dogs were presented to the neurology service of the above-mentioned clinic or external veterinary clinics or practices (Clinic for Small Animals of the University of Leipzig, Veterinary Clinic Lüneburg, Anicura Veterinary Clinic Haar, Veterinary Practice for Neurology in Dettelbach, Clinic for Small Animals of the Ludwigs-Maximilian-University Munich, and Small Animal Clinic Piding) due to neurological signs between June 2014 and February 2022. External CSF and serum samples were sent to the research laboratory for determination of IgA levels in CSF and serum. Samples of the healthy control group originated from the university-owned beagle colony (animal experiment number: 33.8-42502-05-20A561). CSF and serum samples for H3Cit ELISA and DNase activity assays were stored at −80°C. Only residual samples of routine diagnostic work up were analyzed.

Diagnostic workup of the emergency cases included a general and neurological examination, complete blood cell count (CBC), blood chemistry profile, CRP, IgA determination in serum and CSF by ELISA as previously described [17], and CSF examination consisting of leukocyte cell count, cell differentiation and total protein measurement. Further diagnostics in terms of urine analysis, imaging diagnostics consisting of X-ray, computed tomography, or magnetic resonance imaging, and individually requested special examinations were performed depending on clinical signs. If necessary, a histopathological or microbiological examination of a biopsy or cerebrospinal fluid sample was examined to confirm the suspected diagnosis.

Individual patient information such as signalement, medical history, clinical signs, duration and course of disease, hematologic and clinical chemistry findings, results of CSF analysis and findings of imaging diagnostics were retrospectively collected using the easyvet patient management system (easyvet; VetZ GmbH; Isernhagen; Germany) or were obtained from the submitting veterinary clinics or veterinary practices on personal request. A complete list of selected patient data and summary of all available findings can be found in Table 1 for CSF samples used for IF and TEM staining or in Tables 2 and 3 used for H3Cit ELISA and DNase activity assay.

**Table 1:** CSF samples of animals for IF-/TEM-staining of NETs in CSF samples.

ID	disease group	breed	age (mo)	sex	WBC/3 μL CSF	WBC/10^3^ μL	IgA CSF (μg/mL)	IgA serum (μg/mL)	IF/TEM
1	SRMA acute	German Shorthaired Pointer	7	m	505	10,1	3,78	345,47	IF
1	SRMA remission	German Shorthaired Pointer	8	m	1	6,41	0,19	n/a	IF
1	SRMA relapse	German Shorthaired Pointer	9	m	113	19,7	0,26	200,46	IF
1	SRMA remission	German Shorthaired Pointer	11	m	1	7,48	0,22	139,95	IF
1	SRMA remission	German Shorthaired Pointer	12	m	3	7,2	0,26	240,85	IF
2	SRMA acute	Münsterländer	18	f	1476	19,1	2,65	400,41	IF
3	SRMA acute	Beagle	7	m	130	19,43	0,24	63,4	IF
3	SRMA remission	Beagle	9	m	8	10,45	0,12	244,43	IF
4	SRMA acute	Boxer	7	m	338	17,76	1,73	122,7	IF
5	SRMA acute	Labrador Retriever	11	m	422	25,1	0,91	426,35	TEM

CSF: cerebrospinal fluid, f: female, ID: patient identification, IF: immunofluorescence microscopy, IgA: Immunoglobulin A, m: male, mo: month, μg: microgram; μL: microliter, mg: milligram, mL: milliliter, n/a: not applicable, SRMA: steroid-responsive meningitis-arteritis, TEM: transmission electron microscopy, WBC: white blood cell count

WBC: 6 x 10^3^/μL to 12 x 10^3^/μL, WBC CSF: <5 leukocytes/μL, IgA CSF: <0,2 μg/mL, IgA serum: 10,9 to 100,1 μg/mL

**Table 2:** CSF and serum samples for H3Cit ELISA and DNase activity assay.

ID	disease group	breed	age (mo)	Sex	WBC /3μL CSF	WBC × 10^3^/μL	Neutr. Gran. × 10^3^/μL	IgA CSF (μg/mL)	IgA serum (μg/mL)	TP (mg/dL)
**1**	Bacterial encephalitis	German Shepherd Dog	19	m	27392	18,69	14,8	n/m	28,42	1972,88
**2**	Bacterial encephalitis	Maltese	68	fn	1760	12,49	10,02	1,01	82,02	144,5
**3**	Bacterial. encephalitis	American Pitbull	132	mn	18	5,80	n/a	2,19	494,85	39,6
**4**	Meningioma	Mix	120	m	17	85,90	6,07	0,37	140,54	19,74
**5**	Meningioma	Golden Retriever	132	fn	324	9,76	7,15	0,26	64,44	44,54
**6**	Meningioma	French Bulldog	120	m	18	9,48	n/a	0,98	410,33	44,82
**7**	Meningioma	Elo	72	fn	26	8,11	n/a	3,13	100,23	61,21
**8**	control	Beagle	n/a	f	0	6,09	3,99	n/a	n/a	13,99
**9**	control	Beagle	n/a	m	1	7,34	5,1	n/a	n/a	12,37
**10**	control	Beagle	n/a	f	3	6,93	4,75	n/a	n/a	14,17
**11**	control	Beagle	n/a	f	1	4,96	3,06	n/a	n/a	12,95
**12**	control	Beagle	n/a	f	0	5,23	2,76	n/a	n/a	10,36
**13**	control	Beagle	n/a	f	0	6,23	3,44	n/a	n/a	10,42
**14**	SRMA acute	Sloughi	12	f	229	15,10	12,05	0,2	129,79	20,77
**15**	SRMA acute	Golden Retriever	16	m	2404	21,77	14,32	2,02	150,25	138,72
**16**	SRMA acute	Giant Schnauzer	10	fn	9500	24,27	19,17	13,28	119,45	200,96
**17**	SRMA acute	Weimaraner	7	m	3650	20,18	15,44	0,4	99,56	52,87
**18**	SRMA acute	German Shorthaierd Pointer	7	f	505	10,10	8,41	3,78	345,47	61,04
**19**	SRMA acute	Münsterländer	18	m	1476	19,10	15,17	2,65	400,41	52,2
**20**	SRMA acute	Beagle	7	m	130	19,43	13,0	0,24	63,4	20,72
**21**	SRMA acute	Labrador Retriever	11	m	422	21,31	15,53	0,91	426,35	37,28
**22**	SRMA acute	Boxer	7	m	448	17,76	14,79	1,73	122,70	255,93
**23**	SRMA acute	Mix	12	f	800	26,45	n/a	n/m	223,84	70,4
**24**	SRMA acute	Boxer	13	m	120	26,64	19,71	1,73	122,7	93,2
**25**	SRMA acute	Noca Scotia Duck Tolling Retriever	20	f	n/a	29,20	23,54	n/m	320,56	217,5
**26**	SRMA acute	Weimaraner	23	f	n/a	30,10	23,39	0,61	87,75	87,0
**27**	SRMA acute	German Longhaired Pointer	21	m	n/a	14,07	9,68	2,69	56,4	162,3
**28**	SRMA acute	Bernese Mountain Dog	19	f	n/a	29,29	20,73	2,78	1482,88	66,8
**29**	SRMA acute	Labrador Retriever	12	f	549	7,91	5,28	8,3	129,50	100,0
**30**	SRMA acute	Boxer	18	f	4064	20,72	17,31	0,45	456,8	500,0
**31**	SRMA acute	Mix	12	m	6288	33,43	26,35	4,8	142,12	942,0
**32**	SRMA acute	Boxer	10	f	128	12,00	9,57	6,04	272,99	100
**33**	SRMA acute	German Shorthaierd Pointer	27	f	824	13,31	11,77	5,12	144,21	100
**34**	SRMA acute	Mix	22	m	4128	22,0	17,71	11,59	451,16	500
**35**	SRMA acute	Hungarian Shephard Dog	13	f	498	25,95	20,33	0,36	252,97	30
**36**	SRMA acute	Boxer	n/a	f	7168	27,30	n/a	1,73	122,7	n/a
**37**	SRMA acute	Magyan Vizsla	n/a	m	17900	25,1	n/a	6,23	500,85	n/a
**38**	SRMA acute	Boxer	n/a	m	3250	n/a	n/a	0,87	110,44	n/a
**39**	SRMA acute	Mix	n/a	m	230	n/a	n/a	1,42	84,54	n/a
**40**	SRMA acute	Nova Scotia Duck Tolling Retriever	19	f	74	29,3	26,66	0,2	133,43	15
**41**	SRMA acute	Beagle	15	f	225	14,4	10,8	2,37	619,83	53
**42**	SRMA acute	Beagle	11	f	677	n/a	n/a	4,71	177,3	93
**43**	SRMA acute	American Bulldog	10	f	2997	n/a	n/a	5,15	138,85	275
**44**	SRMA acute	Boxer	18	m	1968	27,4	23,29	n/m	406,92	103
**45**	SRMA acute	Boxer	8	m	1835	27,7	21,6	3,16	383,2	144
**46**	SRMA acute	Beagle	8	f	29	16,9	12,17	1,22	141,58	25
**47**	SRMA acute	German Pinscher	9	f	939	n/a	n/a	0,76	75,19	32
**14**	SRMA therapy	Sloughi	14	f	4	n/a	n/a	0,05	35,03	11,23
**15**	SRMA therapy	Golden Retriever	16	m	1	10,2	7,13	2,02	169,12	12,68
**16**	SRMA therapy	Giant Schnauzer	12	fn	0	9,72	n/a	0,26	26,21	13,33
**17**	SRMA therapy	Weimaraner	9	m	0	13,98	11,31	0,22	98,87	11,69

CSF: cerebrospinal fluid, f: female, fn: female neutered, ID: patient identification, IF: immunofluorescence microscopy, IgA: Immunoglobulin A, m: male, mn: Male neutered, mo: month, μg: microgram; μL: microliter, mg: milligram, mL: milliliter, n/a: not applicable, SRMA: steroid-responsive meningitis-arteritis, TEM: transmission electron microscopy, WBC: white blood cell count

WBC: 6 x 10^3^/μL to 12 x 10^3^/μL, neutrophilic granulocytes: 3 x 10^3^/μL to 10 x 10^3^/μL, WBC CSF: <5 leukocytes/3 μL CSF, IgA CSF: <0,2 μg/mL, IgA serum: 10,9 bis 100,1 μg/mL, TP: >25 mg/dL

**Table 3:** Summary of all patient information and clinical parameters divided into the different disease groups.

disease group	Clinical signs	Median age (year)	Sex	Median WBC /3μL CSF	Median WBC ×10^3^/μL	Median IgA CSF (μg/mL)	Median IgA serum (μg/mL)
**SRMA acute (n=34)**	neck pain (n=27), fever (n=27), hunched posture (n=12), apathy (n=9), inappetence (n=3), stiff gait (n=2), diarrhoe (n=2),	1 year 1 month	m (n=15) f (n=18) fn (n=1)	591	21,3	1,42	141,58
**SRMA therapy (n=4)**	no clinical signs	1	m (n=2) f (n=1) fn (n=1)	1	9,72	0,26	35,03
**meningioma (n=4)**	head tilt (n=3), tetraparesis (n=2), ataxia (n=1), head turn (n=1), abnormal behavior (n=1)	5 years 8 months	m (n=2) fn (n=2)	26	9,5	0,98	140,54
**bacterial. Encephalitis (n=3)**	ataxia (n=2), head turn (n=2), proprioceptive deficits (n=1)	10 years 5 months	m (n=1) mn (n=1) fn (n=1)	1760	12,5	1,6	82,02
**control (n=6)**	no clinical signs	n/a	m (n=1) f (n=5)	1	6,23	n/a	n/a

CSF: cerebrospinal fluid, f: female, fn: female neutered, ID: patient identification, IF: immunofluorescence microscopy, IgA: Immunoglobulin A, m: male, mn: Male neutered, mo: month, μg: microgram; μL: microliter, mg: milligram, mL: milliliter, n/a: not applicable, SRMA: steroid-responsive meningitis-arteritis, TEM: transmission electron microscopy, WBC: white blood cell count

WBC: 6 x 10^3^/μL to 12 x 10^3^/μL, neutrophilic granulocytes: 3 x 10^3^/μL to 10 x 10^3^/μL, WBC CSF: <5 leukocytes/3 μL CSF, IgA CSF: <0,2 μg/mL, IgA serum: 10,9 bis 100,1 μg/mL, TP: >25 mg/dL

The retrospective study population was divided according to previously published diagnostic criteria or guidelines into acute SRMA, SRMA in remission [1, 8, 9, 13, 17], meningioma with concurrent neutrophilic inflammation [63], bacterial encephalitis [64, 65] and healthy control group. Patients were classified into the “SRMA acute group”, if clinical signs and laboratory parameters supported the diagnoses as described in the introduction [1, 8, 9, 13, 17]. The “SRMA in remission group” consisted of dogs currently treated with immunosuppressive therapy after diagnosis of SRMA was made. The dogs had unremarkable clinical and neurologic examinations 6 to 8 weeks after the initial diagnosis and immunomodulatory therapy [1, 8]. Laboratory diagnostic findings supported the remission, such as resolution of neutrophil pleocytosis in the CSF. CSF and serum samples from dogs with suspected meningioma or bacterial encephalitis were included based on clinical signs, magnetic resonance imaging and CSF analysis and diagnostic criteria according to Radaelli et al. [64] and Elbert et al. [65] for bacterial meningoencephalitis and Dickinson [63] for intracranial neoplasia. Animals of the meningioma and bacterial encephalitis group were only included in this study if neutrophilic pleocytosis was present in CSF analysis.

In total 51 CSF and serum samples of dogs were analyzed. CSF and serum samples of n = 34 dogs with acute SRMA and n = 4 dogs with SRMA in remission, n = 4 dogs with meningioma and concurrent neutrophil inflammation, n = 3 dogs with bacterial meningitis and n = 6 healthy dogs were included in this study.

### CSF preparation for immunofluorescence microscopy

CSF processing for IF-microscopy was performed immediately after suboccipital punctuation. 8 mm glass cover slides (Thermo Scientific Fisher; Waltham; Massachusetts; USA) were placed in 48 well multiwell plates (Greiner bio-one 677102; Kremsmünster; Austria) and coated for 20 minutes with 60 μL of 0.01% Poly-L-lysine (Sigma P4707-50ML Sigma-Aldrich; Munich; Germany). 100 μL CSF consisting of a maximum of 2 x 10^5^ cells/100 μL were transferred on these precoated cover slides after taking of the Poly-L-lysine and washing twice with 200 μL of phosphate buffered saline (PBS) (Sigma P5493-1l; 10x PBS diluted to 1x PBS with pyrogen free water; Sigma-Aldrich; Munich; Germany). The plates were centrifuged at 250 x g for 5 minutes at 4°C and fixed in a final concentration of 4% paraformaldehyde (PFA) (Science Services E15710-250; Science Services GmbH; Munich; Germany). The supernatant was carefully removed and stored at −80°C.

### CSF immunofluorescence staining of NETs

Cells fixed on glass slides in 4% PFA were analyzed for NET detection in the CSF compartment of dogs with SRMA. DNA intercalating dyes were used like DAPI to stain the DNA backbone of NETs. NET-specific DNA-histone-1-complexes and cell-specific proteins that are frequently detected in association with NETs such as H3Cit and MPO were stained in colocolization with antibody-based techniques to prove NET-formation [66–69]. Co-staining of DNA-histone-1-complexes and MPO respectively H3Cit was performed according to the protocol previously described [70–72] with the following changes:

After washing the slides three times with 1x PBS they were permeabilized for 5 minutes in 0.5% Triton X-100 (Sigma-Aldrich T8787-50ML; Sigma-Aldrich; Munich; Germany) diluted in PBS and blocked for 20 minutes (blocking buffer for co-staining of DNA-histone-1-complexes and MPO: 1% bovine serum albumin, 3% donkey serum, 3% cold water fish gelatin and 0.05% Tween20 diluted in PBS; blocking buffer for co-staining of DNA-histone-1-complexes and H3Cit: 2% bovine serum albumin, 10% fetal calf serum, 0.05% Tween20 and 0.1% Triton X-100 diluted in PBS). Afterwards, the slides were incubated for one hour with a mouse monoclonal IgG2a anti-DNA/histone (Millipore MAB3864; Billerica; Massachusetts; USA; 2,2 mg/mL; 1:1000) and rabbit anti human myeloperoxidase (Dako; A0398; 3,3 mg/mL; 1:337.5) or rabbit anti-human H3Cit (citrulline R2 + R8 + R17) antibody (Abcam; ab5103; Cambridge; UK; 1 mg/mL, 1:32) diluted in respective blocking buffer. For isotype control murine IgG2a (from murine myeloma M5409; Sigma Aldrich; Munich; Germany; 0.2 mg/mL; 1:100) was used diluted in the respective blocking buffer. Rabbit IgG (from rabbit serum I5006; Sigma Aldrich; Munich; Germany; 1.16 mg/mL) was used in dilutions in the respective blocking buffer of 1:108.75 for staining of MPO and 1:36 for staining of H3Cit.

After washing with PBS, the secondary antibody staining was performed for one hour in the dark at room temperature. The following antibodies were used diluted in respective blocking buffer: goat anti‐rabbit Alexa 633‐conjugated antibody (Invitrogen; Carlsbad; California; USA; 2 mg/mL; 1:500) and a goat anti‐mouse Alexa 488‐conjugated antibody (Invitrogen; Carlsbad; California; USA; 2 mg/mL; 1:500). After washing steps DNA was counterstained and the slides were embedded in ProLong^®^Gold antifade reagent with DAPI (Invitrogen P36930; Carlsbad; California; USA).

### Immunofluorescence microscopy of CSF neutrophils

The stained slides of CSF samples were examined on a Leica TCS SP5 AOBS confocal inverted-base fluorescence microscope with HCX PL APO 40 × 0.75–1.25 oil immersion objectives with diode laser of a wavelength of 405 nm, an Argon-Laser with a wavelength of 488 nm and a Helium-Neon-Laser with a wavelength of 633 nm (Leica; Wetzlar; Germany). The settings were adjusted using isotype control antibodies in separate preparations for each staining. Pictures were edited with ImageJ software (version 1.53n; National Institute of Health; USA).

### Quantification of CSF neutrophils in immunofluorescence images

Four dogs with acute SRMA and one dog with relapsed SRMA were included for the quantification of NETs in the CSF (Table 1). Quantification of the number of netting neutrophils was performed by two methods. The first method was manually counting activated neutrophils with enlarged nuclei or neutrophils actively extruding NETs in the CSF per 387.5 μm x 387.5 μm immunofluorescence image. The number of netting neutrophils was related to the total amount of cells in the image in percent and illustrated in S10 Fig.

The second method was performed by using an immunofluorescence intensity measurement using Image J software (version 1.53n; National Institute of Health; USA). The area of positive H3Cit signal representing NETs was related to the area of the nucleus identified as positive DAPI signal and illustrated in S10 Fig.

### Electron microscopy of CSF neutrophils

CSF processing for transmission electron microscopy (TEM) was performed immediately after suboccipital puncture. A maximum of 1 x 10^6^/100 μL neutrophils were transferred to a 1.5 mL reaction tube and centrifuged at 400 x g for 5 minutes at room temperature. The supernatant was carefully removed and stored at −80°C. The cell pellet was fixed in 250 μL of 2.5% (vol/vol) glutaraldehyde in 0.1 M sodium cacodylate (pH 7.2), post fixed with 1% osmium tetroxide (wt/vol) and 0.15 M sodium cacodylate (pH 7.2) for 1 h at 4°C, washed and further processed for electron microscopy.

For transmission electron microscopy, the fixed and washed samples were subsequently dehydrated in ethanol and further processed for standard Epon 812 embedding as described previously [73]. Images were recorded with a Gatan Multiscan 791 charge-coupled device camera (Gatan Incorporated; Pleasanton; California; USA). The ultrathin sections were stained with uranyl acetate (Laurylab; Saint Fons; France) and lead citrate (Laurylab; Saint Fons; France). Immunolabeling of thin sections after antigen unmasking with sodium metaperiodate (Merck; Darmstadt; Germany) with gold-labeled anti-histone and anti-neutrophil elastase (NE) was performed as described previously [74, 75], with the modification that Aurion-BSA (Aurion; Wageningen; The Netherlands) was used as a blocking agent. Pictures were edited with ImageJ software (version 1.53n; National Institute of Health; USA).

### H3Cit ELISA

The measurement of H3Cit in CSF and serum samples was performed with a Citrullinated Histone H3 (Clone 11D3) ELISA Kit (Cayman Chemical; Ann Arbor; Michigan; USA) according to the manufacturers’ instructions in Multiskan Go microplate reader (Thermo Fisher Scientific; Waltham; MA; USA).

### DNase activity assay

DNase activity in serum and CSF samples was measured with a DNase I Activity Assay Kit (Fluorometric) (Catalog # K429-100; Biovision Incorporated; Milpitas; California; USA) in Tecan Spark Plate Reader (Tecan Group AG; Männedorf; Schweiz) according to the manufacturers’ instructions with the following changes: the excitation/emission (Ex/EM) was measured at a wavelength of 550/670 nm.

### Statistical analysis

Data were analyzed using Microsoft Excel 2007 and 2016 (Microsoft; Albuquerque; New Mexico; USA). Statistical analyses and graphical illustration were performed with GraphPad Prism Version 8.0.1 (San Diego; California; USA) and SAS Enterprise Guide 7.1 (SAS Institute Incorporated; Cary; North Carolina; USA). At first the generated data were analyzed for Gaussian distribution using the Kolmogorov-Smirnov-Test. Because the data of H3Cit-levels were not normally distributed non-parametric tests were used for further analysis. A non-parametric analysis of variance (ANOVA) in terms of a Kruskal-Wallis-Test followed by a Dunn’s multiple comparisons test was performed. The data of the DNase-activity-test were normally distributed so that an ordinary one-way ANOVA was performed followed by Holm-Šidáks multiple comparisons test. A Grubbs´s testing to identify potential outliers was additionally performed (S1 Table).

Afterwards non-parametric correlations between H3Cit levels and DNase-activity in serum and CSF were analyzed using Spearman’s rank correlation coefficient. Further correlations between H3Cit-levels and DNase activity in CSF and serum and parameters like WBC, neutrophilic granulocytes, total protein of the CSF, leukocytes in the CSF [1, 5, 13, 76], and IgA-levels in serum and CSF [17] in the group of SRMA acute diseased dogs were performed using Spearman’s rank correlations coefficient after testing each parameter concerning normal distribution with Kolmogorov-Smirnov-test. Furthermore, the stability of the samples was checked by performing non-parametric correlations using Spearman´s rank correlation coefficient on storage length and ELISA respectively DNase activity assay results (S6–S9 Figs). The figure legends offer a detailed description of tests used to analyze the received data from the H3Cit ELISA and DNase activity test. The *p*-value has the same settings in all graphs (*p < 0.05, ****p < 0.0001).

## Results

### Low-grade amount of NETs could be detected in the CSF of acute diseased dogs using immunofluorescence microscopy

Patients with acute SRMA were confirmed according to the above-mentioned diagnostic criteria. The presence of a low-grade amount of NETs in CSF samples during the acute stage of the disease was confirmed with immunofluorescence staining followed evaluation with confocal immunofluorescence microscopy.

**Fig 1:**
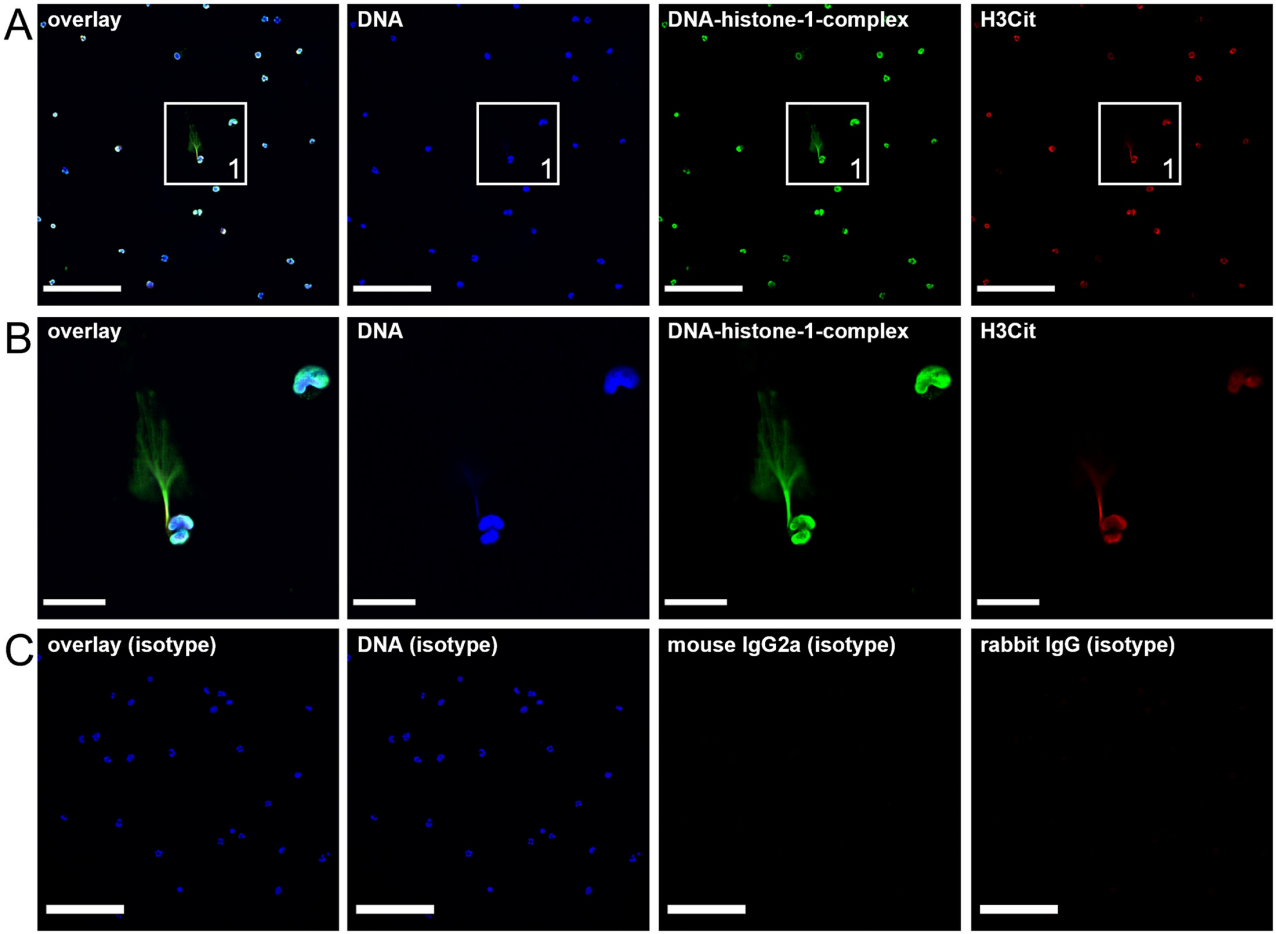
Few amounts of NETs in the CSF of a 7-month-old male German Shorthaired Pointer. **(A)** Neutrophil extracellular traps (NET) as decondensed extracellular DNA-fibers decorated with NET-specific proteins like citrullinated Histone H3 (H3Cit) were detected in the cerebrospinal fluid during acute onset of the disease. Only a few NETs were detected and appear in spiky formations. Blue = counterstaining of DNA (Hoechst), green = DNA/histone-1-complexes (NETs), red = citrullinated histone H3 (H3Cit). Representative images are shown. Scale bar = 100 μm. **(B)** Zoom picture of area 1 is presented showing a singular NET-event consisting of H3Cit combined with DNA-Histon-1-complexes. Blue = DNA (Hoechst), green = DNA/histone-1-complexes (ETs), red = citrullinated histone H3 (H3Cit). Scale bar = 20 μm. **(C)**. Settings of the immunofluorescence images were adjusted to a respective isotype control. Representative images of the respective isotype control are presented. Scale bar = 100 μm.

**Fig 2:**
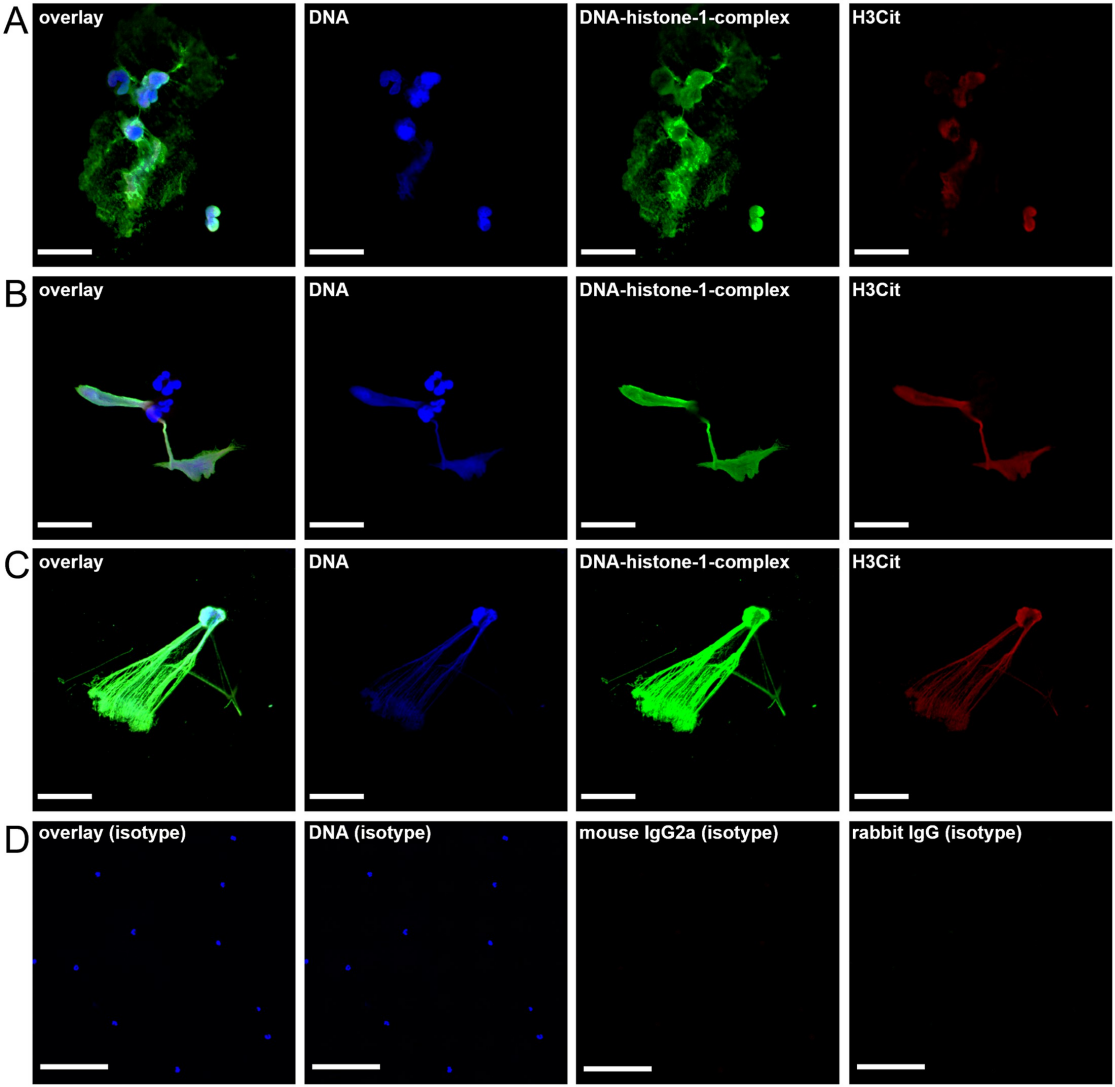
Spiky and cloudy NETs in a 7-month-old female Beagle. **(A)** Neutrophil extracellular traps (NET) as combined extracellular DNA-fibers presented as cloudy NET-formations with staining of NET-specific citrullinated histone H3 (H3Cit) and DNA-histone-1-complexes were detected in the cerebrospinal fluid during acute onset. Blue = counterstaining of DNA (Hoechst), green = DNA/histone-1-complexes (NETs), red = citrullinated histone H3 (H3Cit). Representative images are shown. Scale bar = 100 μm. **(B), (C)** Spiky NET-formations were detected. Blue = DNA (Hoechst), green = DNA/histone-1-complexes (NETs), red = citrullinated histone (H3Cit). Scale bar = 20 μm. **(D)** Settings of the immunofluorescence images were adjusted to a respective isotype control. Respective images of the respective isotype control are presented. Scale bar = 100 μm.

Extracellular NET-structures were visible in three of four dogs during acute onset of SRMA (Figs 1 and 2). The majority of neutrophils do not show any extracellular NET-formation in confocal microscopy or just a small amount of activated nuclei. Only a few neutrophils were decorated with extracellular NETs and appear in spiky (Figs 1A, 1B, 2B and 2C) respectively cloudy (Fig 2A) formations. Other immune cells like very rare spotted macrophages were not surrounded by NETs. 2.7% to 17% of the neutrophils in the images showed visible ET-formation (S10 Fig). The measurement of fluorescence intensity using Image J compared NETs (H3Cit = red) to nucleus (DAPI = blue). 3.3% to 16.8% positive signal of H3Cit was detectable compared to the signal of the nucleus (S10 Fig). Based on these results, we hypothesized, that vesicular NET-formation is possibly the more common and major way of NET-formation in SRMA affected dogs during the acute phase of the disease. Follow up samples taken after 6 weeks of immunosuppressive treatment of 2/4 dogs showed only singular mononuclear cells and no detectable NET-formations surrounding these cells. Follow up of 2/4 dogs was not possible because the patient owners did not approve to another CSF tap.

### Vesicular NETosis was detected in the CSF of one exemplary acute diseased dog using transmission electron microscopy

As a next step glutaraldehyde fixed CSF cell pellet of one exemplary dog affected with acute SRMA was analyzed with TEM to visualize especially ultrastructural processes of vesicular and confirm NET-formation. TEM allows more detailed and especially intracellular analysis of NET-releasing neutrophils *ex vivo* due to higher resolution than confocal microscopy. H3Cit and NE were stained with immunogold-labeling to facilitate the visualization of these NET-specific components in the cytoplasm and cell organelles.

**Fig 3:**
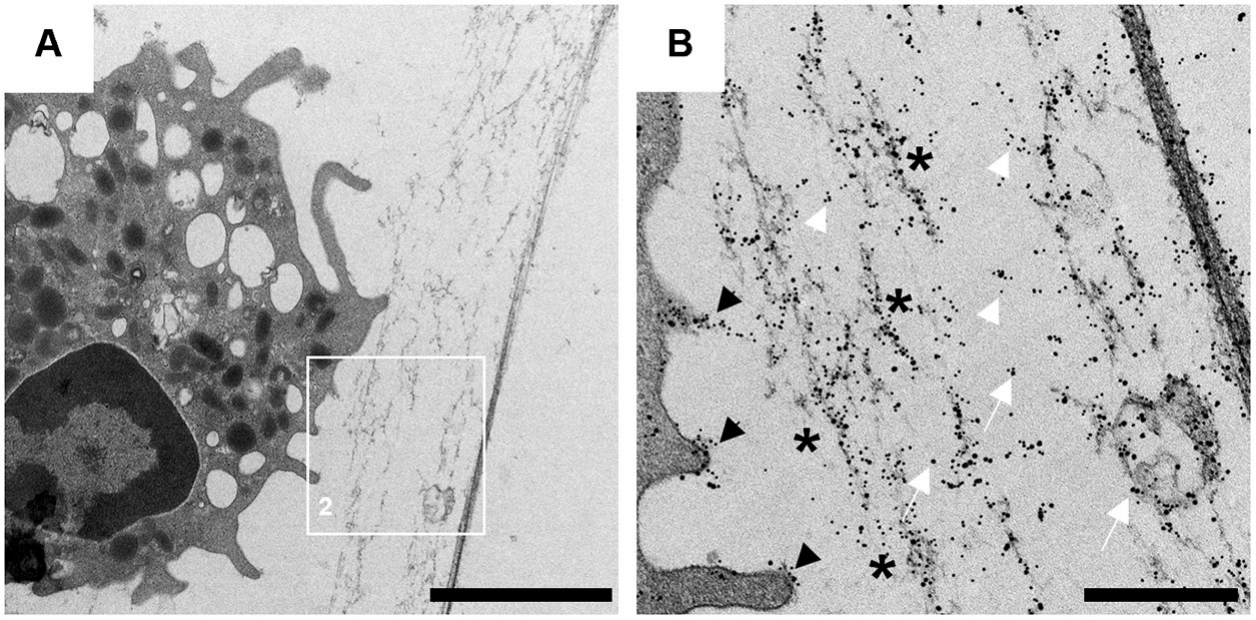
Ultrastructural NET-Formation of an 11-month-old male Labrador Retriever. Representative image (3A) of CSF neutrophils and the respective magnifications (3B) of extracellular NET-Formation in this individual patient were shown in both images. Citrullinated histone H3 (H3Cit) (white arrowhead) and neutrophil elastase (NE) (white arrow) were detected with immunogold-labeling in neutrophil granulocytes taken from CSF samples during acute onset of SRMA (gold/H3Cit = 5 nm, gold/neutrophil elastase = 10 nm). Examination was performed via transmission electron microscopy. Black asterisks show filamentous chromatin decorated with H3Cit and NE representing NET structures (3B). NET-components like H3Cit and NE were budding near the cell membrane forming spiky convexities at the cell membrane and show the release of NETs in the extracellular space (black arrowheads) (3B). Left image (3A): scale bar = 2 μm. Right image (3B): scale bar = 500 nm.

In Fig 3 characteristic signs of extracellular NET-structures are shown. Filamentous chromatin fibers decorated with immunogold-labelled NET-markers H3Cit and NE were released at the cell membrane in the extracellular space forming typical extracellular NETs.

**Fig 4:**
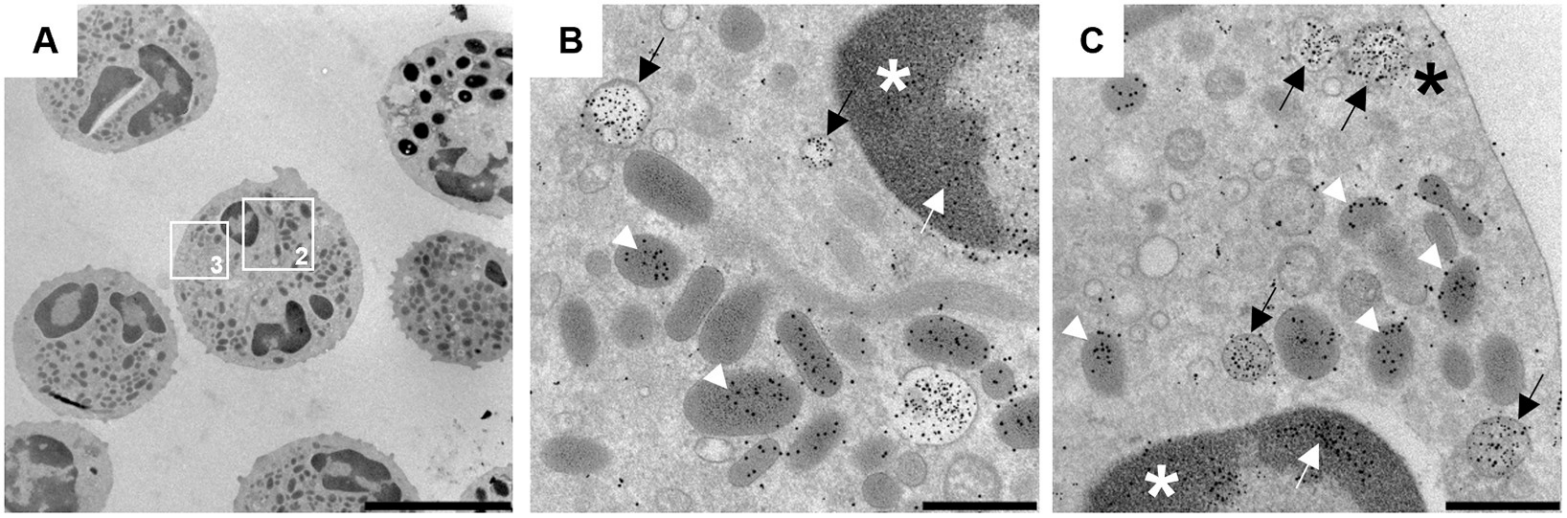
Ultrastructural vesicular NET-Formation in an 11-month-old male Labrador Retriever. Representative images (4A-4C) of CSF neutrophils and respective magnifications of ultrastructural processes of intracellular NET-Formation in this individual patient were shown in each row. Citrullinated histone H3 (H3Cit) and neutrophil elastase (NE) were detected with immunogold-labeling in neutrophil granulocytes taken from CSF samples during acute onset of SRMA (gold/H3Cit = 5 nm, gold/neutrophil elastase = 10 nm). Examination was performed via transmission electron microscopy. NE (white arrows) was present in the nucleus (white asterisk) as definite indicator for early NETosis and catalyst of chromatin decondensation in cooperation with myeloperoxidase (MPO) (4B). Colocalized H3Cit and NE were present with high amounts in multiple, nuclear, light grey vesicles (black arrow) in the cytoplasm in 4B, 4C representing intracellular created NETs. In 4C nuclear vesicles positive for H3Cit and NE were detected near the cell membrane (black asterisk). NE was present in dark grey neutrophil granules (white arrowheads) (4B, 4C). Left column: scale bar = 5 μm. Middle and right column: scale bar = 500 nm.

In Fig 4 and S2 Fig typical signs of intracellular NET-formation are shown. Nuclear vesicles in the cytoplasm were labeled positively for H3Cit and NE. The translocation of NE in the nucleus was detectable in Fig 4B. Translocated NE represents another strong evidence of early NETosis, because NE in correlation with MPO is sufficient for chromatin-decondensation and histone degradation [77]. Furthermore, in Fig 4C neutrophils show budding near the cell membrane to release their NET-containing vesicles–the proof of vesicular NET-formation [33–35, 78]. The majority of neutrophils of this exemplary dog show characteristics of vesicular NETosis.

The intracellular detection of NE and H3Cit serving as NET-specific markers in netting neutrophils of the CSF in SRMA diseased dogs leads to further hypotheses about the pathogenesis of SRMA. NETs are additionally detectable with IF-staining as well as with gold-labelling and TEM. Their influence and role as possible immunologic participant of the complex pathogenesis of SRMA is feasible. Overall ultrastructural portrayal of NETosis by transmission electron microscopy is superior to confocal microscopy due to detection of early steps of NETosis [31, 66].

### Concentration of H3Cit is not significantly elevated in CSF samples of acute diseased dogs suffering from SRMA

**Fig 5:**
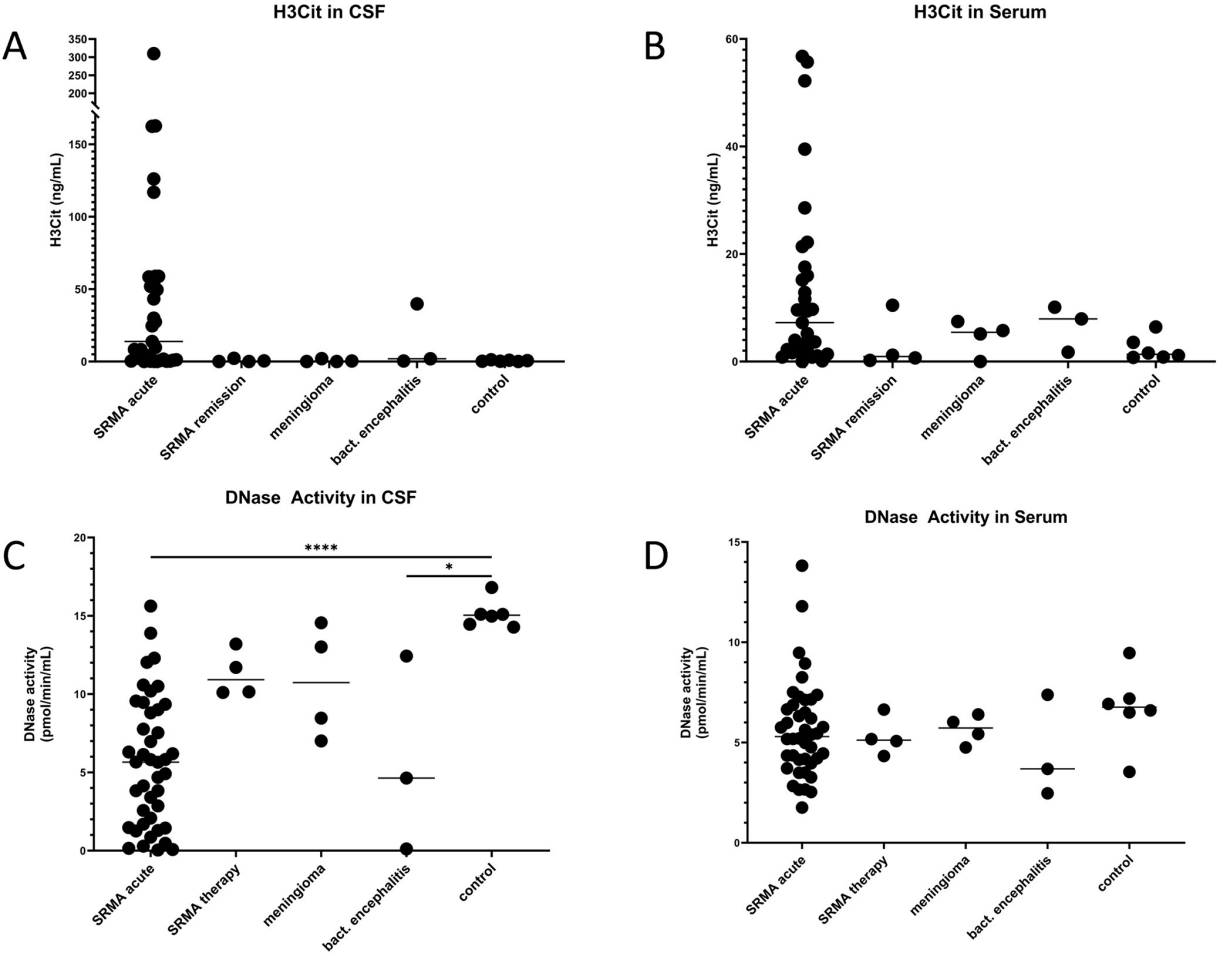
Concentration of H3Cit-levels and DNase activity in CSF and serum samples. Concentration of H3Cit (A, B) and measurement of DNase activity (C, D) in CSF and serum samples of the different disease groups. Lines in between the individual values indicate the respective median. Asterisks represent significant differences between the groups connected through lines above the graph (*p < 0.05, ****p < 0.0001). A non-parametric analysis of variance (ANOVA) (Kruskal-Wallis-Test) concerning citrullinated histone H3 (H3Cit) followed by a Dunn’s multiple comparisons test and an ordinary one way (ANOVA) followed by Holm-Šidáks multiple comparisons test concerning DNase activity was performed due to statistical analysis of the generated data. Bact. encephalitis: bacterial encephalitis; CSF: cerebrospinal fluid; DNase: deoxyribonucleases, mL: milliliter; min: minute; pmol: picomol, SRMA: steroid-responsive meningitis-arteritis.

Levels of H3Cit in serum and CSF samples of dogs were measured using commercial ELISA techniques to investigate quantitative evidence of histone citrullination as indirect marker of NET-formation (Fig 5A and 5B). In n = 2 serum sample and n = 7 CSF samples the H3Cit concentration could not be determined because the H3Cit concentration was outside the measurement range of the ELISA. The highest value of H3Cit was 309.51 ng/mL of one dog suffering from acute SRMA. A description of the median H3Cit concentration and ranges of H3Cit levels in CSF and serum is shown in Table 4.

**Table 4:** Median concentrations and ranges of H3Cit levels (ng/mL) in CSF and serum of different disease groups.

Disease group	Samples (CSF/serum)	Median H3Cit ng/mL CSF	Median H3Cit ng/mL serum	Range H3Cit ng/mL CSF	Range H3Cit ng/mL Serum
**SRMA acute**	34/34	13,84	7,23	0–309,51	0,02–56,75
**SRMA therapy**	4/4	0,23	0,935	0,29–0,87	0,23–10,48
**Meningioma**	4/4	0,195	5,45	0–1,99	0–7,44
**Bacterial encephalitis**	3/3	1,96	7,94	0,39–39,83	1,74–10,1
**control**	6/6	0,41	1,35	0–1,22	0,77–6,43

CSF: cerebrospinal fluid, H3Cit: citrullinated histone H3, μL: microliter, mL: milliliter, ng: nanogram, SRMA: steroid-responsive meningitis-arteritis

Especially in CSF and serum samples of the SRMA acute group several values of different animals are highly elevated compared to the other disease groups and control group. Statistical significance could not be determined between each disease group regardless of serum and CSF samples.

### DNase activity is significantly reduced in dogs suffering from acute SRMA

Host DNases are essential enzymes to control the NET-metabolism, in particular maintaining the balance between perpetual NET-formation and NET-degradation [59, 60]. In all available CSF and serum samples of each different group DNase activity was detectable (Fig 5C and 5D). A description of the median DNase activity and ranges of DNase activity in CSF and serum is shown in Table 5.

**Table 5:** Median concentrations and ranges of DNase activity (pmol/min/mL) in CSF and serum of different disease groups.

Disease group	Samples (CSF/serum)	Median DNase activity pmol/min/mL CSF	Median DNase activity pmol/min/mL serum	Range DNase activity pmol/min/mL CSF	Range DNase activity pmol/min/mL Serum
**SRMA acute**	34/34	5,285	5,175	0,05–13,89	1,76–11,8
**SRMA therapy**	4/4	10,12	4,7	0,86–11,7	2,53–5,17
**Meningioma**	4/4	10,74	5,885	7,01–14,55	5,42–6,49
**Bacterial encephalitis**	3/3	4,36	3,68	0,11–12,43	2,47–7,38
**control**	6/6	15,04	6,765	14,28–16,81	3,54–9,47

CSF: cerebrospinal fluid, DNase: deoxyribonuclease, min: minute, mL: milliliter, ng: nanogram, pmol: picomol, SRMA: steroid-responsive meningitis-arteritis, WBC: white blood cell count

The control group showed the highest activity of DNase activity with a median of 15.1 pmol/min/mL. Statistical significance of DNase activity in the CSF was detected between the SRMA acute group and control group (*p* < 0.0001) and bacterial encephalitis and control group (*p* = 0.0142). In serum samples no statistical significances could be detected comparing each group to another.

### Correlation analysis

In Table 6 the correlation analysis of H3Cit and DNase activity in serum and CSF samples is shown aside from the disease group. H3Cit levels in CSF and H3Cit levels in serum were weak positively associated (*r*_*s*_. = 0.25). DNase activity in serum and H3Cit levels in serum and DNase activity in CSF were weakly negatively associated (*r*_*s*_. = –0.14). DNase activity in CSF and serum are moderate positively correlated (*r*_*s*_. = 0.48). A strong and significant negative association was present between H3Cit concentration in CSF and DNase activity in CSF (*r*_*s*_. = –0.52, *p* = < 0.0001).

**Table 6:** Correlation analysis: Association between H3Cit levels and DNase activity in serum and CSF about all groups.

Parameters	*r*_*s*_	*p*	Interpretation
H3Cit concentration in CSF and H3Cit concentration in serum	0.25	0.0753	+
H3Cit concentration in CSF and DNase activity in CSF	**–0.52**	**<0.0001**	**+++**
H3Cit concentration in CSF and DNase activity in serum	–0.14	0.3362	+
H3Cit concentration in serum and DNase activity in CSF	–0.14	0.3342	+
H3Cit concentration in serum and DNase activity in serum	0.07	0.6511	0
DNase activity in CSF and DNase in serum	0.48	0.0004	++

CSF: cerebrospinal fluid, DNase: deoxyribonucleases, H3Cit: citrullinated histone H3, 0: no correlation (*r*_*s*_: < +/– 0.1), +: weak correlation (*r*_*s*_: +/– 0.1–0.3), ++: moderate correlation (*r*_*s*_: +/– 0.3–0.5), +++: strong correlation (*r*_*s*_: > +/– 0.5). Bold printed values show significance (*p* < 0.0001). The parameters were analyzed using Spearman´s rank correlation coefficient.

In Table 7 the correlation analysis of H3Cit and DNase activity in serum and CSF samples is shown for the SRMA acute group. A moderate negative association was present between H3Cit concentration in CSF and DNase activity in CSF (*r*_*s*_. = –0.30) and a moderate positive association was shown between DNase activity in CSF and DNase activity in serum (*r*_*s*_. = 0.38). H3Cit levels in serum and DNase activity in CSF (*r*_*s*_. = 0.16) as well DNase activity in serum (*r*_*s*_. = 0.23) were weakly positively associated.

**Table 7:** Correlation analysis: Association between H3Cit levels and DNase activity in serum and CSF in SRMA acute group.

Parameters	*r*_*s*_	*p*	Interpretation
H3Cit concentration in CSF and H3Cit concentration in serum	0.05	0.7906	0
H3Cit concentration in CSF and DNase activity in CSF	–0.30	0.0917	++
H3Cit concentration in CSF and DNase activity in serum	–0.09	0.6201	0
H3Cit concentration in serum and DNase activity in CSF	0.16	0.3704	+
H3Cit concentration in serum and DNase activity in serum	0.23	0.1923	+
DNase activity in CSF and DNase in serum	0.38	0.0252	++

CSF: cerebrospinal fluid, DNase: deoxyribonucleases, H3Cit: citrullinated histone H3, 0: no correlation (*r*_*s*_: < +/– 0.1), +: weak correlation (*r*_*s*_: +/– 0.1–0.3), ++: moderate correlation (*r*_*s*_: +/– 0.3–0.5), +++: strong correlation (*r*_*s*_: > +/– 0.5). Bold printed values show significance (*p* < 0.0001). The parameters were analyzed using Spearman´s rank correlation coefficient.

In Table 8 the correlation analysis of H3Cit and DNase activity in serum and CSF samples was associated with typical elevated laboratory parameters in acute SRMA affected dogs like leukocytes per 3 μL of CSF, white blood cell count (WBC), neutrophil granulocytes in plasma, IgA in CSF and serum, and total protein (TP) in CSF. Leukocytes/3μL in CSF and H3Cit levels in CSF (*r*_*s*_. = 0.52) as well as total protein in CSF (*r*_*s*_. = 0.55, *p* = < 0.0001) were strongly positively associated. Leukocytes/3μL CSF and DNase activity in CSF of the SRMA acute group (*r*_*s*_. = –0.69, *p* = < 0.0001) and TP in CSF and DNase activity in CSF (*r*_*s*_. = –0.61, *p* = < 0.0001) were significantly negatively associated. The correlation analysis of blood neutrophil granulocytes and H3Cit in CSF (*r*_*s*_. = 0.37) and serum (*r*_*s*_. = 0.35) was moderately positively correlated and on the opposite the comparison of neutrophil granulocytes and DNase activity in CSF (*r*_*s*_. = –0.46) and serum (*r*_*s*_. = –0.26) was negatively correlated. IgA values in CSF are positively associated with H3Cit levels in CSF (*r*_*s*_. = 0.48) as well as IgA values in serum are positively associated with H3Cit-levels in serum (*r*_*s*_. = 0.38). The mean storage time of all samples was 24.66 months with a standard deviation of 25.86 months. It is relevant to mention that storage time corelates with H3cit in CSF, but not in serum. However, samples with varying storage times were included in all groups (S6–S9 Figs).

**Table 8:** Correlation analysis: Association between H3Cit levels and DNase activity in CSF and serum and different parameters in SRMA acute.

Parameters	*r*_*s*_	*p*	Interpretation
Leucocytes/3μL CSF and H3Cit CSF	0.52	0.0002	+++
Leucocytes/3μL CSF and H3Cit serum	0.40	0.0065	++
Leucocytes/3μL CSF and DNase activity CSF	**–0.69**	**<0.0001**	**+++**
Leucocytes/3μL CSF and DNase activity serum	–0.13	0.3877	+
WBC and H3Cit CSF	0.47	0.0013	++
WBC and H3Cit serum	0.24	0.1091	+
WBC and DNase activity CSF	**–0.57**	**<0.0001**	**+++**
WBC and DNase activity serum	–0.29	0.0531	++
Neutrophil and H3Cit CSF	0.37	0.0263	++
Neutrophil and H3Cit serum	0.35	0.0347	++
Neutrophil and DNase activity CSF	–0.46	0.0040	++
Neutrophil and DNase activity serum	–0.26	0.1095	+
IgA CSF and H3Cit CSF	0.48	0.0015	++
IgA CSF and H3Cit serum	0.40	0.0089	++
IgA CSF and DNase activity CSF	–0.30	0.0600	++
IgA CSF and DNase activity serum	0.11	0.4997	+
IgA Serum and H3Cit CSF	0.23	0.1178	+
IgA Serum and H3Cit serum	0.38	0.0105	++
IgA Serum and DNase activity CSF	–0.01	0.9496	0
IgA Serum and DNase activity serum	0.18	0.2339	+
TP CSF and H3Cit CSF	**0.55**	**<0.0001**	**+++**
TP CSF and H3Cit serum	0.40	0.0066	++
TP CSF and DNase activity CSF	**–0.61**	**<0.0001**	**+++**
TP CSF and DNase activity serum	–0.11	0.4428	+

CSF: cerebrospinal fluid, DNase: deoxyribonucleases, H3Cit: citrullinated histone H3, 0: no correlation (*r*_*s*_: < +/– 0.1), +: weak correlation (*r*_*s*_: +/– 0.1–0.3), ++: moderate correlation (*r*_*s*_: +/– 0.3–0,5), +++: strong correlation (*r*_*s*_: > +/– 0.5). Bold printed values show significance (*p* < 0.0001). The parameters were analyzed using Spearman´s rank correlation coefficient.

## Discussion

Previous studies described the pathogenesis of SRMA without detecting an explicit trigger leading to this disease [7, 8]. SRMA, a well described natural occurring large animal model, represents a common, most likely immune-mediated disorder with prominent aetiopathogenetic involvement of neutrophilic granulocytes [9]. In a previous study by Wohlsein et al. [79] the proof of principle of NET-visualization in typical, histopathological altered localizations of cervical meninges and cervical respectively peripheral arteries of chronic recurrent cases of SRMA was investigated. Based on these first findings of NETs in the canine CNS and especially in connection with histopathological changes of patients with chronic, recurrent SRMA, this following study wants to visualize NETs and quantify NET-markers in clinical applicable samples like CSF and serum of acute diseased dogs. NET-formation may interdigitate with many known mechanisms of the specific pathogenesis as previously described [79]. This study offers the proof of occurrence and possible contribution of NETs to the pathogenesis of SRMA using four different detection methods in *ex vivo* samples to present strong evidence for their possible pathogenetic involvement in this particular disease.

Three of four CSF samples of dogs with acute SRMA and neutrophilic pleocytosis showed singular to low-grade extracellular NET-release with immunofluorescence technique. The majority of neutrophils were not surrounded by any signals for DNA-histone-1-complexes, H3Cit or MPO in the CSF. The quantification of the images by using the mean fluorescence intensity or manually quantification could underline this finding that only 2.7% to 17% of the neutrophils showed activated nuclei or extracellular NET-structures. Co-staining of DNA-histone-1-complexes and H3Cit respectively MPO with counterstaining of DNA with DAPI is a highly reliable method for NETs-visualization [66–69]. These results of NET-formation in fresh CSF samples confirm the outcome of the previous study detecting NETs in histopathological samples of chronic and recurrent cases of SRMA [79]. We could not differentiate which immune cells are responsible for NET-formation since simulation of in vivo conditions are not possible. We hypothesized that NETs surrounding neutrophils are generated by these explicit cells and are not built by other immune cells. Release of DNA traps was described not only in neutrophils, but also in macrophages in humans [80], goats [81], and cattle [82]. More recently, CD8+ and CD4+ cells were added to the list of cells releasing extracellular DNA traps [83–86]. The majority of cells in CSF analysis and IF-microscopy were neutrophils, so that the origin of extracellular traps is most probably associated with neutrophils–therefore we called them “neutrophil extracellular traps” NETs in the manuscript.

In general, extracellular NETosis is underdiagnosed due to the restricted resolution of confocal microscopy [66]. Based on this finding, a hypothesis can be constructed explaining the present NET-morphology in acute SRMA. The exact localization of NET-formation during the process of leptomeningeal inflammation and leukodiapedesis is unknown. NETs could be released in the inflamed cervical or peripheral arteries and the extracellular chromatin-structure is pulled off the cell body after neutrophil leukodiapedesis through the blood brain barrier. Therefore, NETs are not attached to the neutrophil cell body anymore and could explain possibly the detection of only few amount of NET-events surrounding CSF neutrophils.

On the other hand, NETs could be diluted in the CSF and washed away during laminar flow in the subarachnoid space or after suboccipital puncture. In addition, the exact time point of the occurrence of NET formation during the inflammatory process in SRMA patients is unknown. The presented images of the CSF neutrophils were possibly taken shortly before or after the majority of neutrophils perform NET-formation respectively digestion of NETs was almost done. However, using electron microscopy we could confirm that NET-formation in the CSF of one exemplary diseased dog with SRMA is mainly dominated by vesicular NET-formation.

Furthermore, the onset of the disease, recognition of the symptoms by the owners, possible NET-influencing drugs administered by first-opinion veterinarians, timepoint of presentation at our hospital, and timepoint of analysis of the PFA fixed cells were different for every dog. Nevertheless, three out of four analyzed CSF samples of acutely diseased dogs with SRMA showed cloudy or spiky NET-formation. For the authors’ information and knowledge this is the first study presenting pictures of *ex vivo* NET-formation in the canine CSF. In general, this leads to the assumption that NETs were created in the subarachnoid space during the acute stage of disease and possibly contribute to the pathogenesis of SRMA as hypothesized previously [79]. After 6 weeks of immunosuppressive therapy and evaluation of treatment response performing follow up CSF tap and blood work of 2/4 dogs, NETs were not detectable anymore.

Because the resolution of immunofluorescence microscopy is limited to distinction of early stages of NETosis between activated and non-activated neutrophils respectively nuclei, TEM was used to catch intracellular, ultrastructural processes of vesicular NETosis [66, 87]. Different pathways of NETosis need different methods for visualization. Vital respectively vesicular NETosis is potentially the more common way of neutrophils releasing NETs during the acute phase of SRMA in contrast to suicidal NETosis with release of various shaped, extracellular conformations of NETs. In addition, neutrophils may show a higher percentage of vesicular NETosis than suicidal NETosis in the CSF.

Regardless of different pathways of NET-formation, products of NET-metabolism were secreted in body fluids and raised awareness in clinical studies in human medicine serving as auspicious biomarker in several diseases like sepsis, pancreatitis, cancer, and stroke [55–57, 88]. Our results demonstrate a distinguished involvement of NETs in terms of measurable NET-markers in acute SRMA. We used H3Cit as the most specific marker for NETs as quantitative approach to prove and measure NET-formation [55–58].

Because of its specificity for NET metabolism, H3Cit was in the center of our interest to determine whether this protein is elevated in SRMA and was compared to other infectious or non-infectious inflammatory or non-inflammatory CNS diseases with involvement of neutrophil inflammation (e. g. meningioma with accompanied inflammation). H3Cit levels in CSF and serum samples of several dogs were elevated in the SRMA acute group in comparison to healthy control dogs. Despite that the statistical analysis revealed no significance between the disease groups, the results and distribution of the values indicate a higher number of netting neutrophils in the subarachnoid space of a part of acute diseased dogs in comparison to healthy control group or currently treated dogs with immunosuppressive medication.

In comparison to immunofluorescence microscopy, H3Cit measurement is more practicable for a large number of samples and could be a helpful tool in the future to investigate NET-formation in canine inflammatory neuropathies. H3Cit CSF levels could be developed as a biomarker in future studies. The range of H3Cit levels in CSF was very high up to 309.51 ng/mL in comparison to the range of serum levels up to 56.75 ng/mL. This finding underlines the unique immune compartmentalization of the CSF and the specific involvement of the inflammation of the subarachnoid space.

DNase activity measurement in dogs suffering from SRMA revealed significant lower activity in CSF than in the healthy control group (*p* < 0.0001). A primary or secondary reduced DNase activity could possibly offer a good explanation for deregulated NET-metabolism in the CSF compartment of diseased dogs. The exact mechanism of reducing the DNase activity still must be elucidated. Maybe a comparable pathomechanism like in systemic Lupus erythematosus (SLE) is present and responsible for the reduced DNase activity [59, 89]. In SLE the inhibition of DNase 1 bears on specific DNase 1-inhibitors or SLE-specific antibody-dependent blocking DNase 1 substrates that prevents NET-structures from DNase digestion [59]. This additional *ex vivo* assay supports the theory of an irregular NET-metabolism especially in the CSF compartment of SRMA affected dogs. In addition, bacterial encephalitis and meningioma with concurrent neutrophilic involvement do show slightly reduced DNase activity without statistical significance. This observation has to be further investigated with larger number of individuals in each group, whether inflammatory involvement of neutrophils is negatively associated with DNase activity.

The correlation analysis underlines the causality between increased H3Cit levels and decreased DNase activity in CSF and serum samples of acute diseased dogs with moderate negative association. The main conclusion of the correlation analysis including H3Cit and DNase activity is the following: acute diseased dogs with SRMA possibly suffer from an insufficient NET-clearance possibly caused by reduced DNase activity. The impaired NET-metabolism and NET-forming neutrophils result in elevated H3Cit levels in the CSF compartment.

Furthermore, the representative intrathecal and systemic neutrophil recruitment during the acute phase of SRMA could explain the positive association between leukocytes in the CSF, WBC, and consequently neutrophil granulocytes in the blood and H3Cit levels [1, 3, 19]. On the other way round, leukocytes in the CSF, WBC and consequently neutrophilic granulocytes are negatively associated with DNase activity underlining the main outcome of this study that acute diseased dogs with SRMA suffer from reduced capacity to cleave NETs. The definitive cause of the irregular working DNase 1 in coincidence with elevated NET-formation in SRMA stays obscure.

In addition, further breed specific and genetic studies are required to determine, that SRMA patients have an increased capacity to release NETs like it is known for patients with SLE [41, 59]. A decreased clearance capacity of NETs may promote the immune-mediated stimulation and DNase activity in general needs further investigation in predisposed breeds frequently suffering from SRMA to elucidate a potential genetic reason for insufficient NET-clearance. Individual and breed specific research on the ability to provoke NET-formation in the most prevalent life section of young age could support the theory of a potential genetic condition.

The included dogs in the SRMA acute group showed typical laboratory alterations like elevated IgA levels in CSF and serum as well as neutrophilic pleocytosis of the CSF. IgA levels and total protein concentration in CSF are strongly positively associated to H3Cit levels in CSF. This connection could be well explained by the damaged blood brain barrier in the acute phase of the disease and consequently protein influx of the periphery in the CSF compartment [1, 13].

Otherwise, the intrathecal immunoglobulin, interleukin and chemokine synthesis is elevated in the acute stage of the disease as described earlier and results in elevated protein levels [90]. As hallmark of SRMA IgA levels are significantly elevated in CSF and serum [17]. In the context of NET-formation in SRMA, permanently elevated IgA-levels could provoke neutrophil granulocytes to induce the externalization of NETs as described in human otitis media [91], viral infections [92] as well as *in vitro* studies [93]. IgA levels are elevated over a prolonged period in SRMA cases [8]. This elongated elevation may cause the ongoing stimulation of neutrophil granulocytes releasing NETs, and potentially resulting in an inflammatory response that may leading the clinically observed recurrences in SRMA.

**Fig 6:**
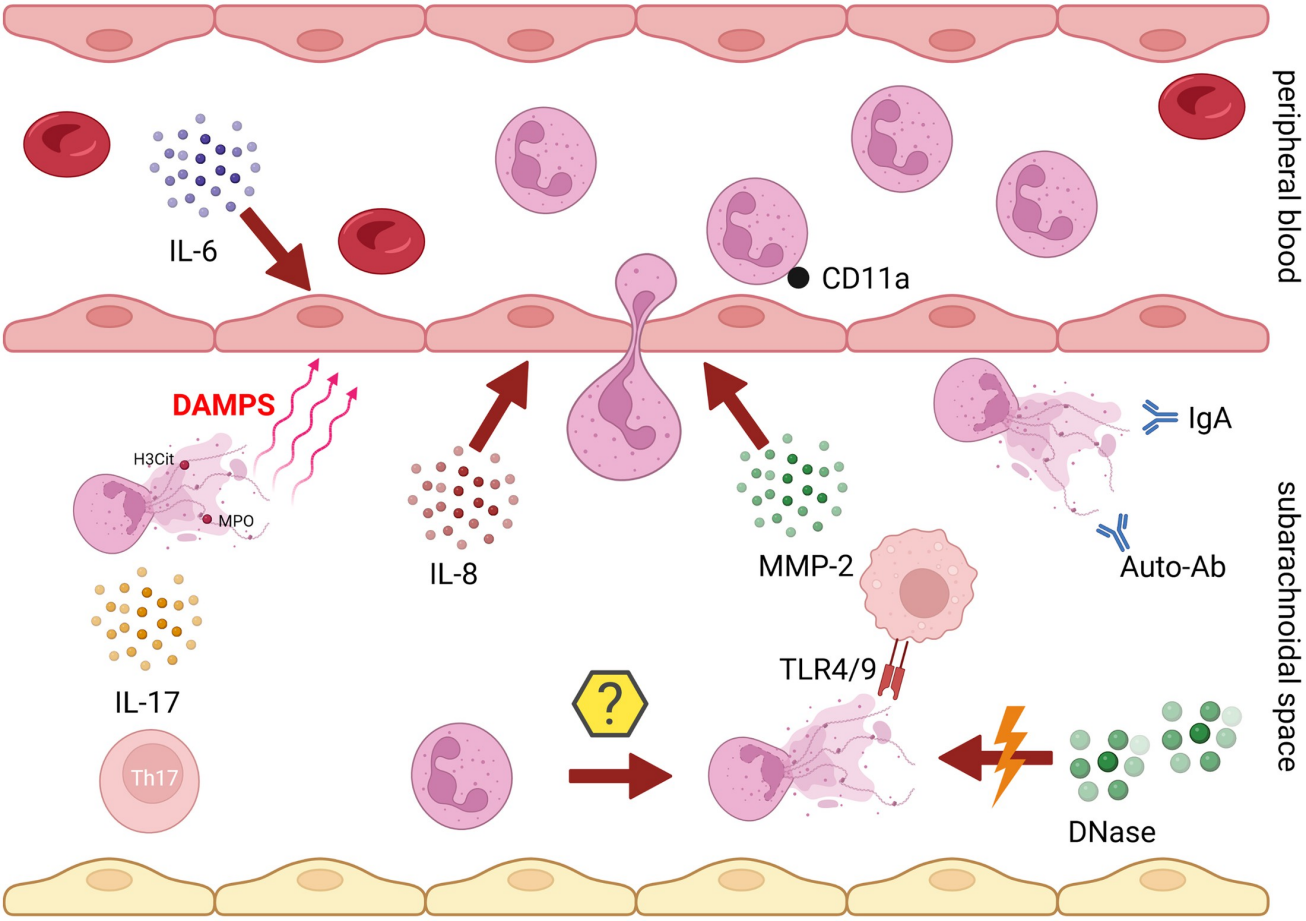
Potential involvement of NETs in the pathogenesis of SRMA.

Furthermore, NET-structures serve as potential source to produce autoantibodies and potentiate the elevated protein levels in the CSF [94]. In addition, total protein and DNase activity in CSF are strongly and significantly negative associated. The assumption of a possible interference between a secreted DNase inhibitor among the elevated total protein content and DNase 1 is feasible. Further studies are necessary investigating serum and CSF of acute diseased dogs regarding a potential DNase inhibitor. A potential involvement of NETs in the pathogenesis of SRMA is illustrated in Fig 6.

Recruitment of neutrophil granulocytes: The leukodiapedesis of neutrophil granulocytes is stimulated by elevated secretion of Interleukin-8 (IL-8) [21] and Interleukin-6 (IL-6) [22]. Increased production of matrix metalloproteases 2 (MMP-2) [20] and upregulation of cluster of differentiation 11a on neutrophilic granulocytes (CD11a) [16] facilitate the crossover across the blood-brain barrier. Neutrophil granulocytes are possibly stimulated by interleukin-17 [IL-17] producing Th17-cells to generate neutrophil extracellular traps (NETs) [23]. The damage-associated proteins (DAMPs) generated from NETs, such as citrullinated histones H3 [H3Cit] or myeloperoxidase (MPO), may interact with Toll-like receptor 4 (TLR-4) and may contribute to the maintenance of cervical inflammation and blood-brain barrier damage [26]. NET metabolism is significantly insufficient and presumably impaired in SRMA-affected dogs because of reduced DNase activity in the cerebrospinal fluid. NETs may further serve as autoantigens and produce autoantibodies (auto-Ab). These autoantibodies may lead to the production of additional NETs [94]. Also, permanently elevated intrathecal IgA levels could trigger continuous NET formation [91–93] (created with BioRender.com by JCW).

Despite that, SRMA is promisingly treatable with immunosuppressive agents like glucocorticosteroids [8], relapse (29.4–47.5%) and mortality rates (4.6–8.1%) are demanding an improvement of therapeutic outcome [9, 95–97] especially in the background of undesirable therapeutic side effects. Specific immunosuppressive treatment is lacking in veterinary medicine [98]. NET-targeting drugs suggest an auspicious therapeutic opportunity treating immune mediated in NET-associated diseases [99]. Monoclonal antibodies, NET-cleaving or NET-inhibiting therapeutics are available in human medicine [99] and need to be tested in veterinary medicine or need pharmaceutical approval for therapeutic use.

SRMA is a well described and explored large animal model for neutrophilic meningitis or vasculitis like Kawasaki-Disease in human medicine [23, 26, 62] and could serve collaboratively in a translational research context for launching new therapeutics. Recombinant DNases (e. g. Dornase alfa) are pharmaceutically approved and currently used for thrombolytic treatment [100, 101], cystic fibrosis [102] or during infection with severe acute respiratory syndrome Coronavirus Type 2 (SARS-CoV2) [103–105]. Patients may benefit from administration of recombinant DNases during the acute onset of the disease while a predominant occurrence of neutrophilic granulocytes is present. Administered DNase possibly supports the irregular NET-metabolism in these patients leading to a better therapeutic outcome and quick improvement of clinical signs. Additionally, remnants of NETs retain proinflammatory activity and have to be cleaved after DNase digestion [106]. Application of recombinant DNases may support and improve the clinical outcome of acute diseased dogs suffering from SRMA.

Among various PAD isoforms that have been described, only PAD2 and PAD4 have been shown to be involved in generation of nuclear citrullinated histone H3. Neutralization of CitH3 is discussed as a promising therapeutic strategy for associated detrimental diseases associated with NET-formation e.g., septic shock [107]. As an example, peptidylarginine deiminase 4 inhibitor Chloramidine (Cl-amidine) prevents histone citrullination [108] and is used in mouse models for immune-mediated diseases with proven involvement of irregular NET-metabolism like systemic lupus erythematosus [109] or rheumatoid arthritis [110]. This NET-specific drug needs further consideration to alternative therapy options in the treatment of SRMA in future studies.

## Conclusion

In conclusion, NETs are visualizable in CSF samples of acute diseased dogs suffering from SRMA using IF and TEM. H3Cit as specific marker for NET-formation is elevated in CSF samples of acute affected dogs with SRMA in combination with significantly reduced DNase activity. However, the exact immunologic mechanisms of NET-formation in CSF and CNS tissue of acute diseased dogs with SRMA are not entirely understood. The pathogenetic interactions need detailed research to capture the immunologic response leading to a better understanding of the disease. An insufficient and dysregulated NET-metabolism is present in dogs suffering from SRMA and could possibly explain and complement the complex immunologic dysregulation leading to SRMA. The detection of NETs in SRMA offer many possibilities to explore the existence and aetiopathogenetic influence of this defence mechanism of the innate immune system in infectious and non-infectious canine neuropathies. In addition, this study offers future possibilities investigating the use of NET-markers or aetiopathogenetic influence of NETs in other canine neuropathies. Specific immunotherapy for the treatment of autoimmune mediated diseases is required to overcome unspecific glucocorticosteroids therapy with many undesirable side effects to improve the patients and patient’s owners’ quality of life.

## Supporting information

S1 Fig Few numbers of spiky NETs in a 7-month-old male Boxer. **(A)** Neutrophil extracellular traps (NET) as combined extracellular spiky DNA-fibers were detected in the cerebrospinal fluid during acute onset consisting of NET-specific citrullinated histone H3 (H3Cit) and DNA-histone-1-complexes. Blue = counterstaining of DNA (Hoechst), green = DNA/histone-1-complexes (ETs), red = myeloperoxidase (MPO). Representative images are shown. Scale bar = 20 μm. **(B), (C)** Spiky NET-formations were frequently detected. Blue = DNA (Hoechst), green = DNA/histone-1-complexes (NETs), red = citrullinated histone H3 (H3Cit). Scale bar = 100 μm. **(D)** Settings of the immunofluorescence images were adjusted to a respective isotype control. Respective isotype control is presented. Scale bar = 100 μm.
(TIF)

S2 Fig 11-month-old male Labrador Retriever with SRMA. Representative images (**A1**, **B1**) of CSF neutrophils and respective magnifications of ultrastructural processes of intracellular NET-Formation in this individual patient were shown in each row. Citrullinated histone H3 (H3Cit) and neutrophil elastase (NE) were detected with immunogold-labeling in neutrophil granulocytes taken from CSF samples during acute onset of SRMA (gold/H3Cit = 5 nm, gold/neutrophil elastase = 10 nm). Examination was performed via transmission electron microscopy. NE (white arrows) was present in the nucleus as definite indicator for early NETosis and catalyst of chromatin decondensation in cooperation with myeloperoxidase (MPO) (**A2**, **B2**). Colocalized H3Cit and NE were present with high amounts in multiple, nuclear, light grey vesicles (black arrow) in the cytoplasm in **A2**, **A3**, **B2**, **B3** representing intracellular created NETs. NE was present in dark grey neutrophil granules (white arrowheads) (**A2**, **A3**, **B2**, **B3**). Left column: scale bar = 5 μm. Middle and right column: scale bar = 500 nm.
(TIF)

S3 Fig Correlation analysis: Association between H3Cit levels and DNase activity in serum and CSF about all groups. (TIF)

S4 Fig Correlation analysis: Association between H3Cit levels and DNase activity in serum and CSF in SRMA acute group. (TIF)

S5 Fig Correlation analysis: Association between H3Cit levels and DNase activity in serum and CSF and different parameters in SRMA acute group. (TIF)

S6 Fig Correlation analysis: Association between H3Cit levels in CSF and storage length of the samples. (TIF)

S7 Fig Correlation analysis: Association between H3Cit levels in serum and storage length of the samples. (TIF)

S8 Fig Correlation analysis: Association between DNase activity in CSF and storage length of the samples. (TIF)

S9 Fig Correlation analysis: Association between DNase activity in serum and storage length of the samples. (TIF)

S10 Fig Quantification of NET-formation in acute diseased or relapsed dogs with SRMA. (A) The amount of netting neutrophils was counted manually and was compared to the total amount of nucleated cells in the CSF of four dogs with acute SRMA and one dog with relapse. Four pictures of every animal were included. (B) The mean fluorescence activity of NETs (H3Cit = red channel) was compared the nucleus (DAPI = blue channel) of four dogs with acute SRMA and one dog with relapse. Four pictures of every animal were included.
(TIF)

S1 Table Results H3Cit ELISA and DNase activity assay. (DOCX)

## Abbreviations

ANOVA: analysis of variance
°C: Degree Celsius
CBC: complete blood cell count
CNS: Central nervous system
CRP: C-reactive protein
CSF: Cerebrospinal fluid
DAPI: 4‘, 6-Diamidino-2-Phenylindol
dL: Deciliter
DNA: Deoxyribonucleic acid
DNase: Deoxyribonuclease
ELISA: Enzyme-linked immunosorbent assay
f: female
fn: female neutered
H3Cit: Citrullinated histone H3
ID: identification number
IF: immunofluorescence microscopy
IgA: Immunoglobulin A
IL: Interleukin
m: male
mg: Milligram
mL: Milliliter
mm: Millimeter
MMP: Matrix metalloproteases
mn: male neutered
Mo: months
MPO: Myeloperoxidase
NE: Neutrophil elastase
NET: Neutrophil extracellular traps
nm: Nanometer
pmol: Picomol
PR3: Proteinase 3
SARS-Cov2: severe acute respiratory syndrome Coronavirus type 2
SLE: systemic Lupus erythematosus
SRMA: Steroid-responsive meningitis-arteritis
TEM: transmission electron microscopy
WBC: white blood cell count
μL: Microliter
