# Supplementary figures and images for: Neutrophil extracellular traps in CSF and serum of dogs with steroid-responsive meningitis-arteritis
Source: PLoS One. 2024 Jan 19;19(1):e0295268. doi: 10.1371/journal.pone.0295268 (PMC10798544; doi:10.1371/journal.pone.0295268)

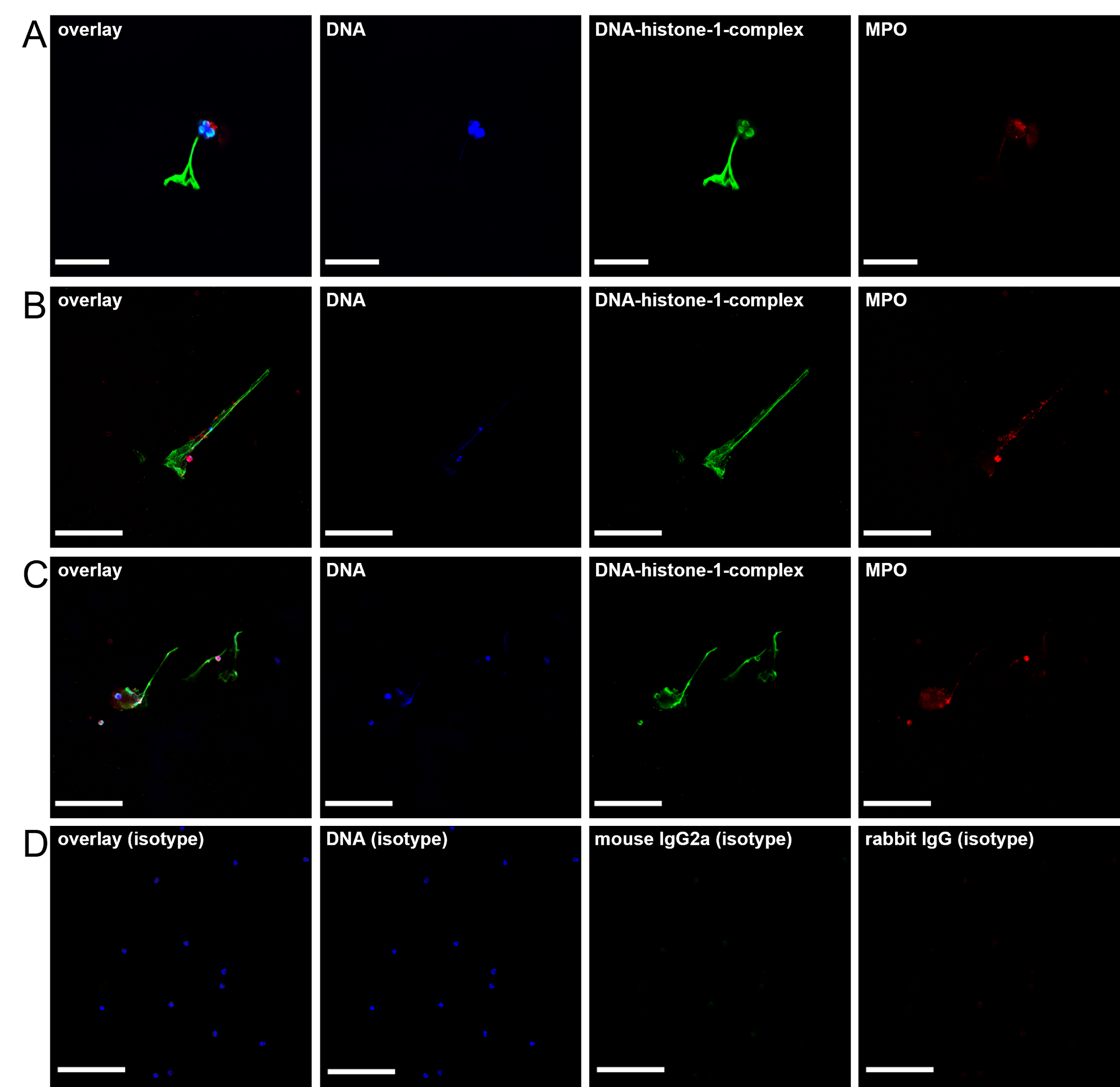

Supplement: S1 Fig — (A) Neutrophil extracellular traps (NET) as combined extracellular spiky DNA-fibers were detected in the cerebrospinal fluid during acute onset consisting of NET-specific citrullinated histone H3 (H3Cit) and DNA-histone-1-complexes. Blue = counterstaining of DNA (Hoechst), green = DNA/histone-1-complexes (ETs), red = myeloperoxidase (MPO). Representative images are shown. Scale bar = 20 μm. (B), (C) Spiky NET-formations were frequently detected. Blue = DNA (Hoechst), green = DNA/histone-1-complexes (NETs), red = citrullinated histone H3 (H3Cit). Scale bar = 100 μm. (D) Settings of the immunofluorescence images were adjusted to a respective isotype control. Respective isotype control is presented. Scale bar = 100 μm. (TIF) [file pone.0295268.s001.tif]

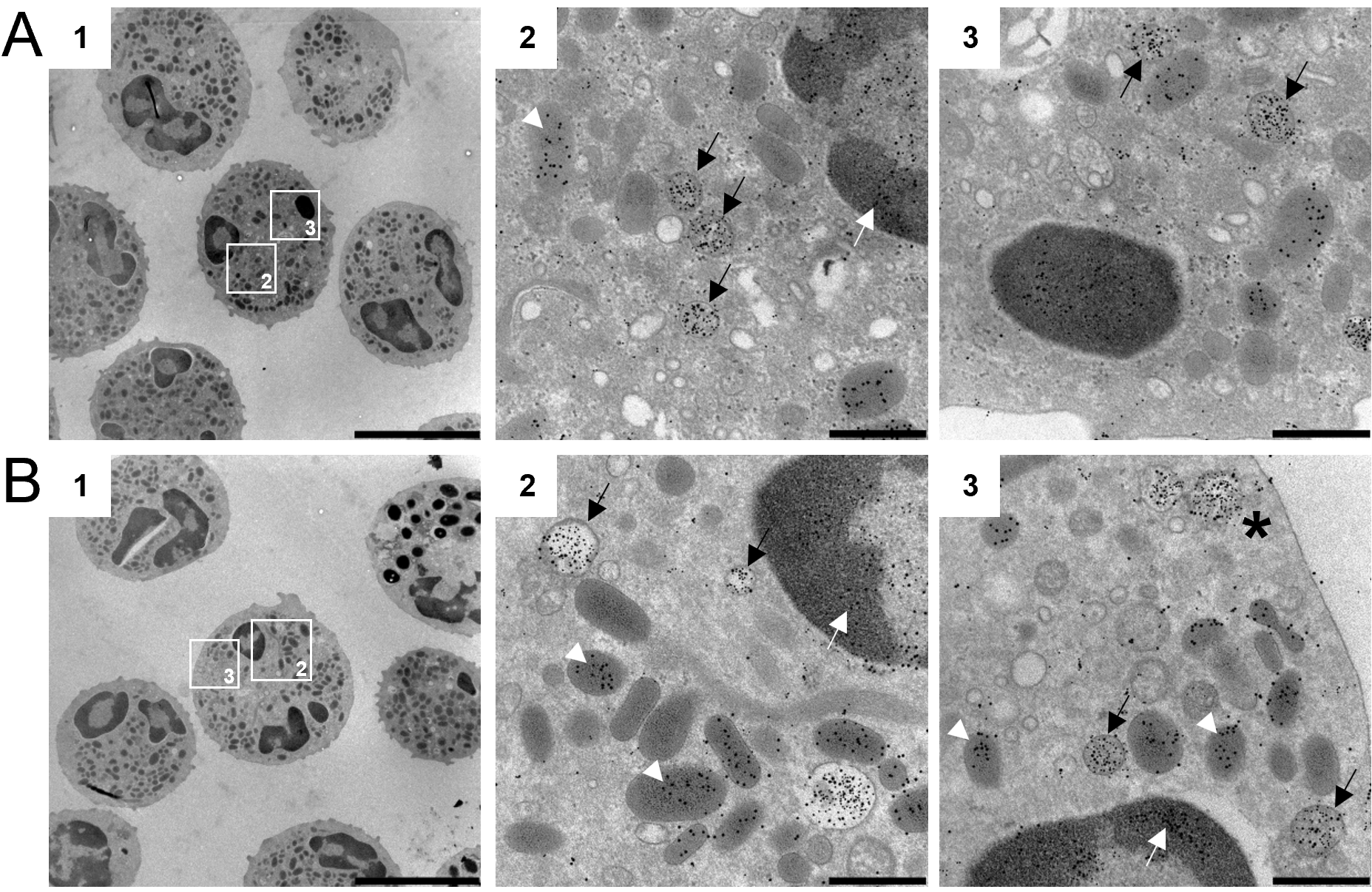

Supplement: S2 Fig — Representative images (A1, B1) of CSF neutrophils and respective magnifications of ultrastructural processes of intracellular NET-Formation in this individual patient were shown in each row. Citrullinated histone H3 (H3Cit) and neutrophil elastase (NE) were detected with immunogold-labeling in neutrophil granulocytes taken from CSF samples during acute onset of SRMA (gold/H3Cit = 5 nm, gold/neutrophil elastase = 10 nm). Examination was performed via transmission electron microscopy. NE (white arrows) was present in the nucleus as definite indicator for early NETosis and catalyst of chromatin decondensation in cooperation with myeloperoxidase (MPO) (A2, B2). Colocalized H3Cit and NE were present with high amounts in multiple, nuclear, light grey vesicles (black arrow) in the cytoplasm in A2, A3, B2, B3 representing intracellular created NETs. NE was present in dark grey neutrophil granules (white arrowheads) (A2, A3, B2, B3). Left column: scale bar = 5 μm. Middle and right column: scale bar = 500 nm. (TIF) [file pone.0295268.s002.tif]

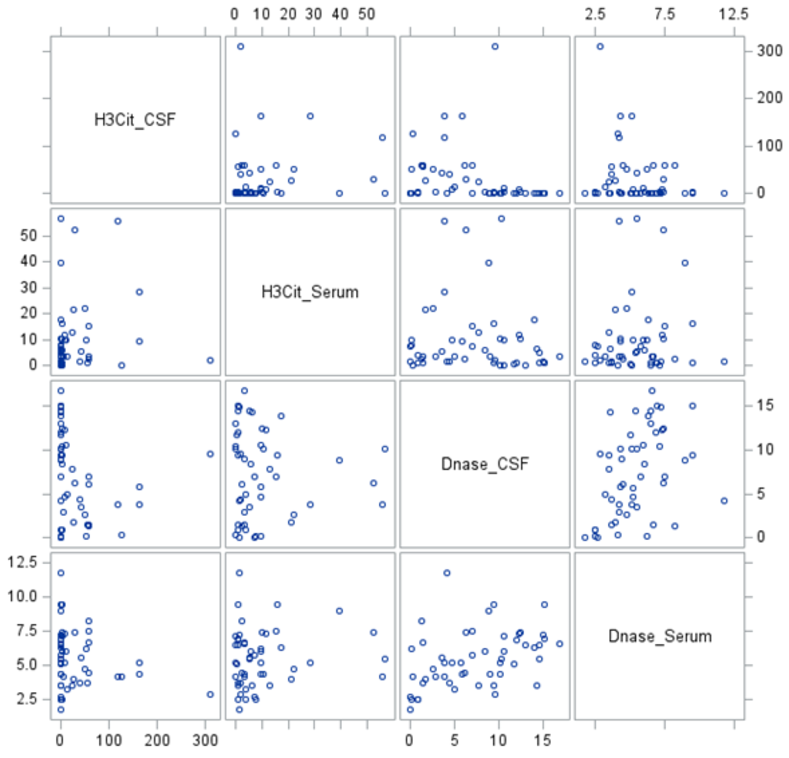

Supplement: S3 Fig — (TIF) [file pone.0295268.s003.tif]

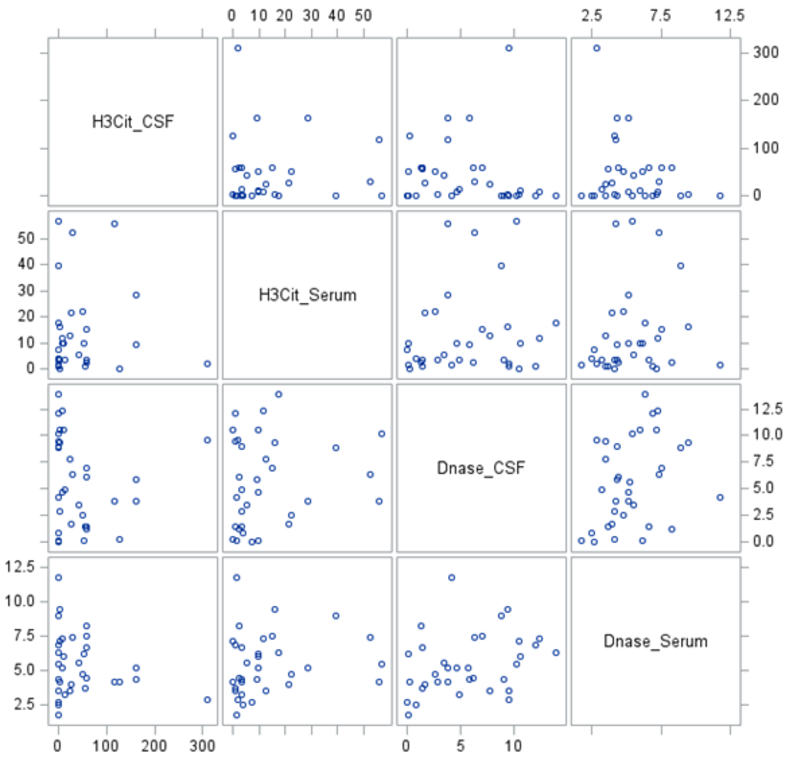

Supplement: S4 Fig — (TIF) [file pone.0295268.s004.tif]

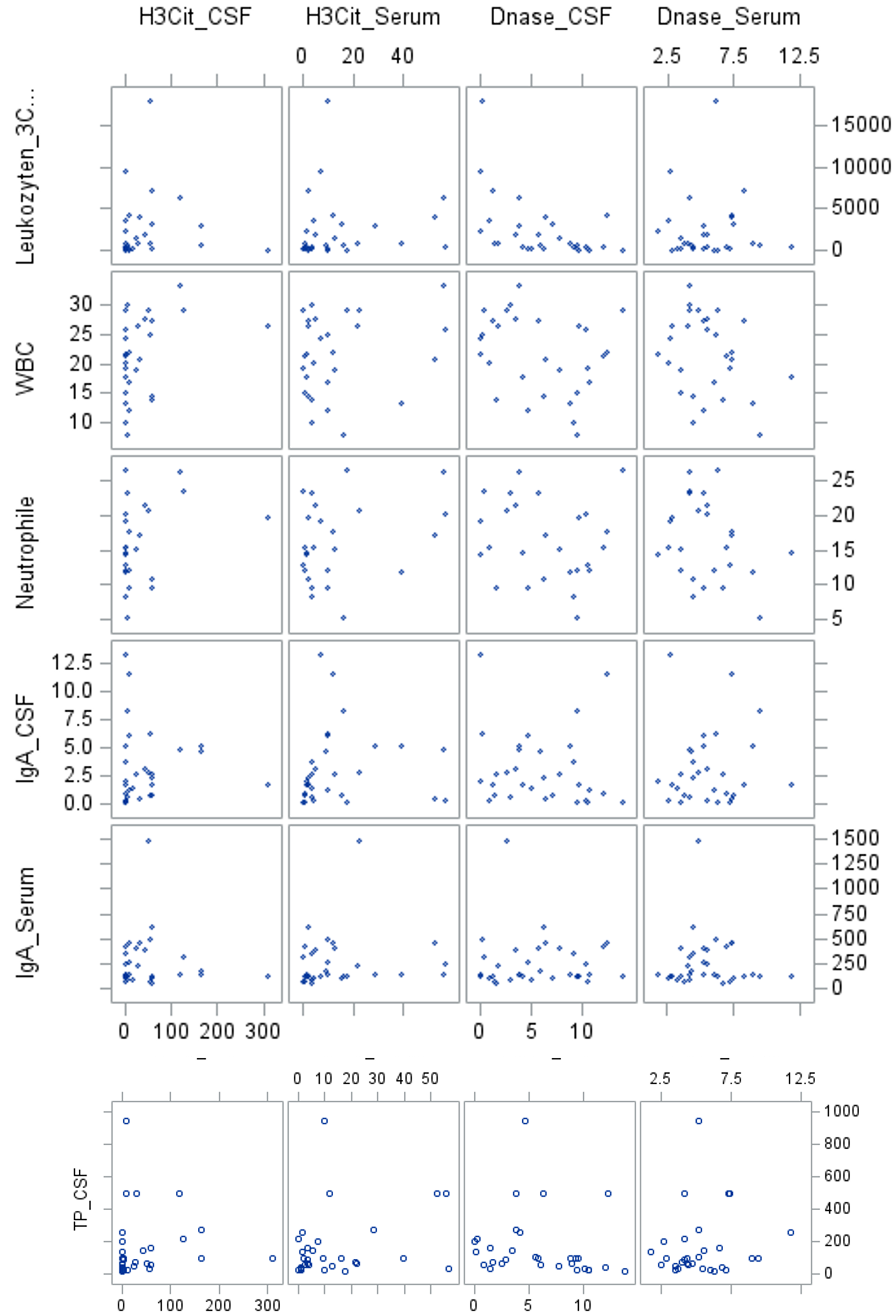

Supplement: S5 Fig — (TIF) [file pone.0295268.s005.tif]

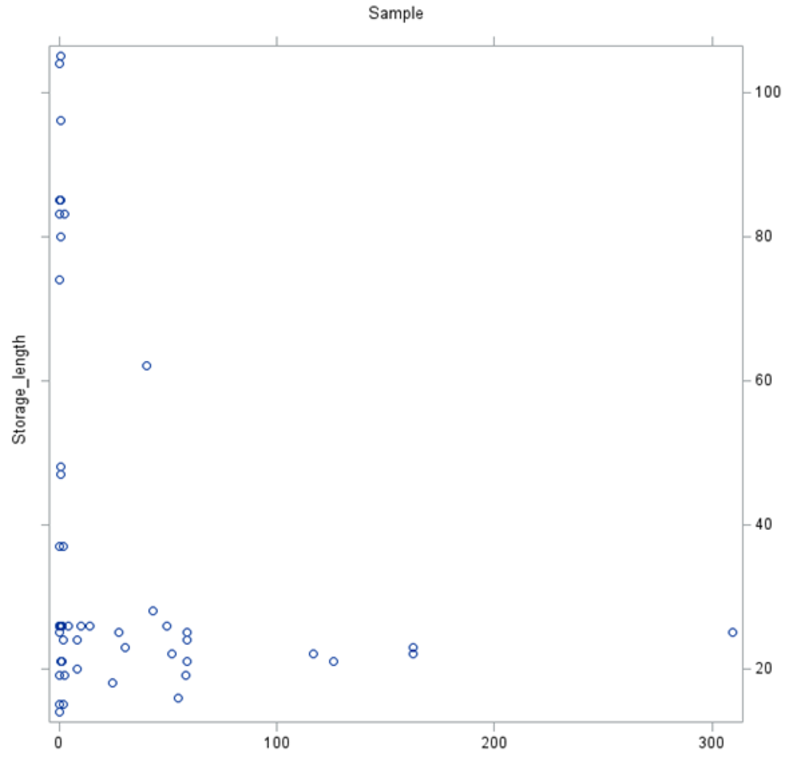

Supplement: S6 Fig — (TIF) [file pone.0295268.s006.tif]

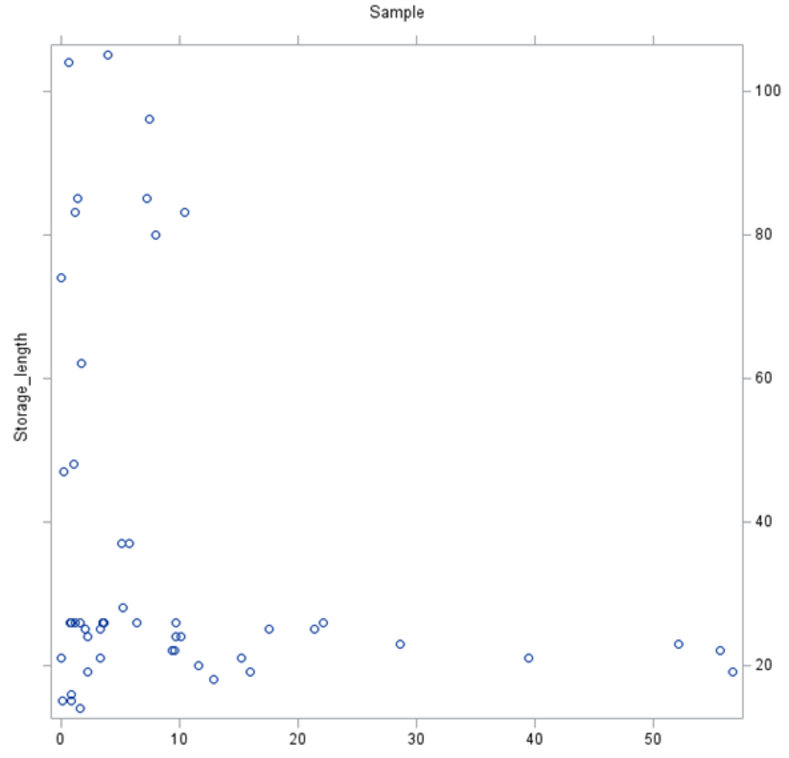

Supplement: S7 Fig — (TIF) [file pone.0295268.s007.tif]

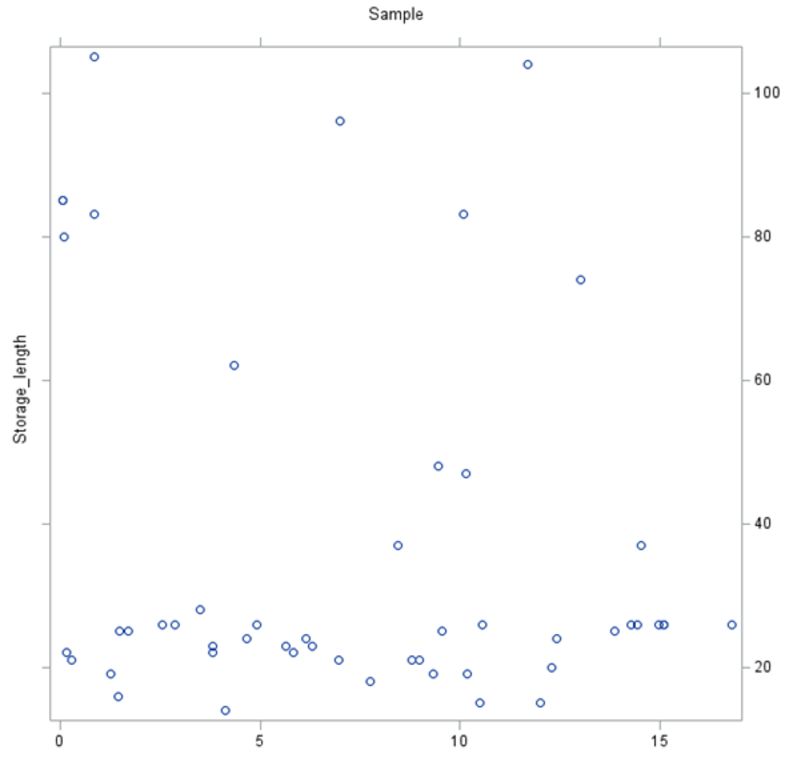

Supplement: S8 Fig — (TIF) [file pone.0295268.s008.tif]

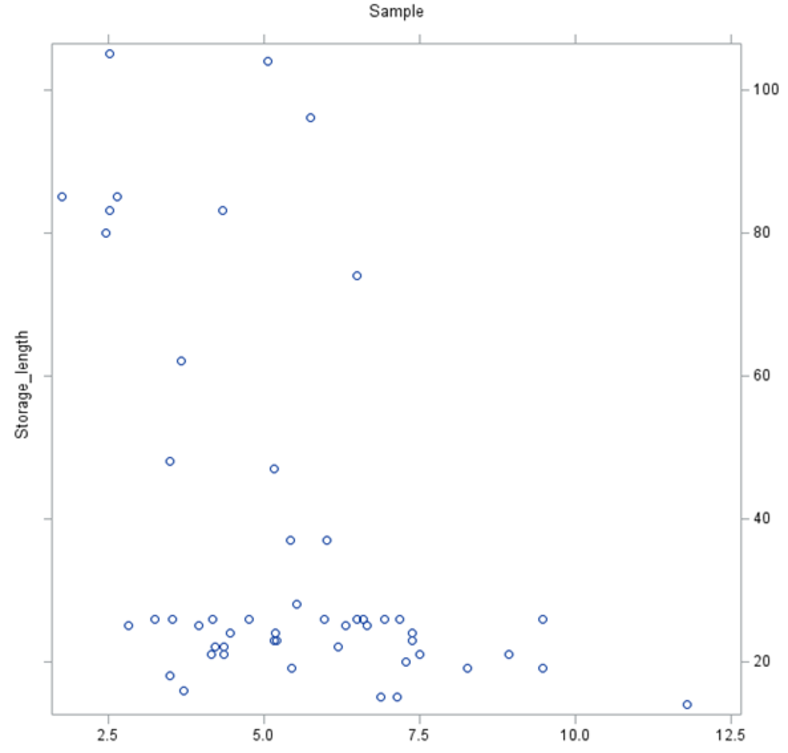

Supplement: S9 Fig — (TIF) [file pone.0295268.s009.tif]

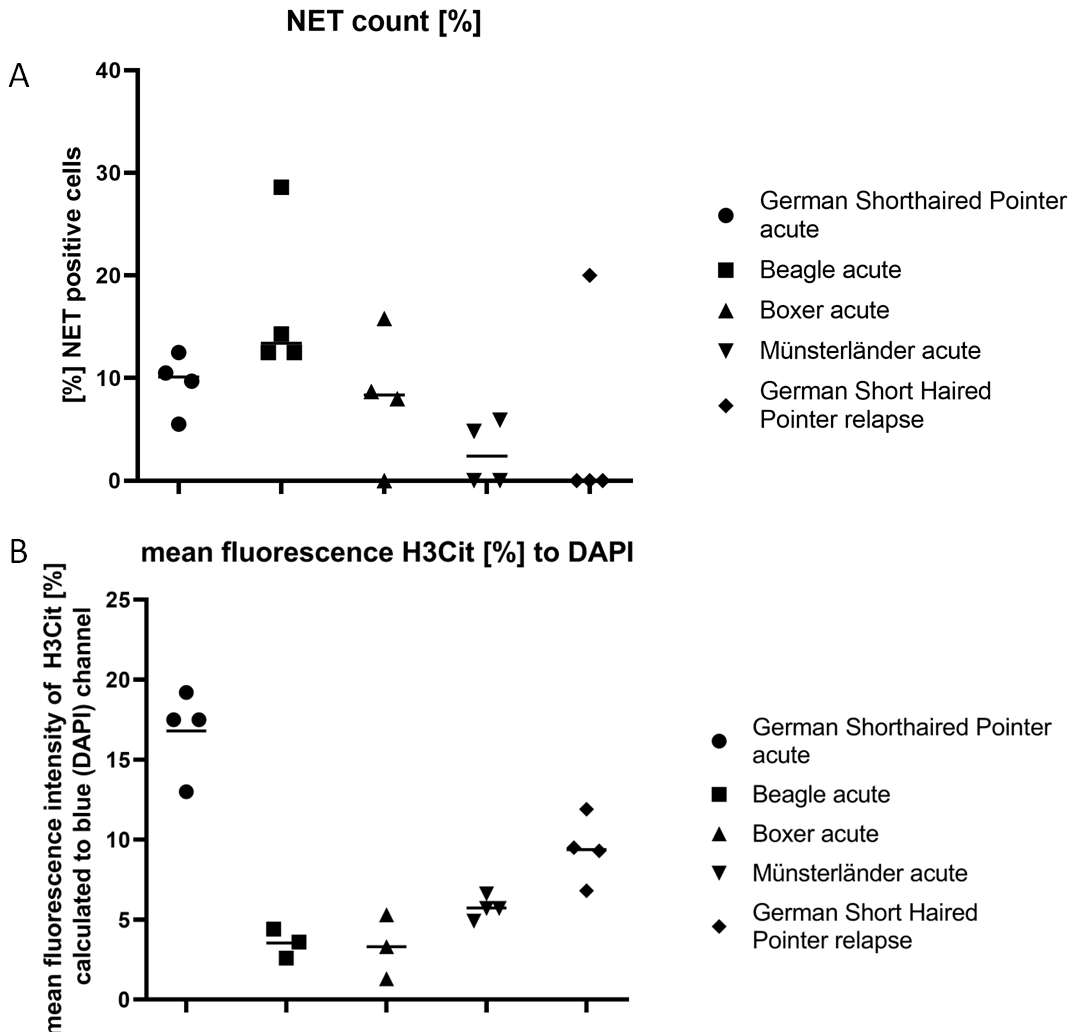

Supplement: S10 Fig — (A) The amount of netting neutrophils was counted manually and was compared to the total amount of nucleated cells in the CSF of four dogs with acute SRMA and one dog with relapse. Four pictures of every animal were included. (B) The mean fluorescence activity of NETs (H3Cit = red channel) was compared the nucleus (DAPI = blue channel) of four dogs with acute SRMA and one dog with relapse. Four pictures of every animal were included. (TIF) [file pone.0295268.s010.tif]
