# Supplementary material for: Neutrophil extracellular traps in CSF and serum of dogs with steroid-responsive meningitis-arteritis
Source: PLoS One. 2024 Jan 19;19(1):e0295268. doi: 10.1371/journal.pone.0295268 (PMC10798544; doi:10.1371/journal.pone.0295268)
Supplement: S1 Table — (DOCX) [file pone.0295268.s011.docx]

**Results of H3Cit-ELISA and DNase activity assay.**

**Supplemental Table 1: Results H3Cit ELISA and DNase activity assay.**

| **ID** | **disease group** | **H3Cit (ng/mL)** | | **DNase-activity (pmol/min/mL)** | | **leukocytes/**  **3 µL CSF** | **WBC**  **x 10^3^/µL** |
| --- | --- | --- | --- | --- | --- | --- | --- |
|  |  | **CSF** | **serum** | **CSF** | **serum** |  |  |
| **1** | bacterial encephalitis | 39,83 | 1,74 | 4,36 | 3,68 | 27392 | 18,69 |
| **2** | bacterial encephalitis | 0,39 | 7,94 | 0,11 | 2,47 | 1760 | 12,49 |
| **3** | bacterial encephalitis | 1,96 | 10,1 | 12,43 | 7,38 | 18 | 5,80 |
| **4** | meningioma | 0,39 | 7,44 | 7,01 | 5,75 | 17 | 8,59 |
| **5** | meningioma | 0 | 0 | 13,02 | 6,49 | 324 | 9,76 |
| **6** | meningioma | 1,99 | 5,76 | 8,46 | 6,02 | 18 | 9,48 |
| **7** | meningioma | 0 | 5,14 | 14,55 | 5,42 | 26 | 8,11 |
| **8** | control | 0,63 | 1,58 | 14,98 | 7,19 | 0 | 6,09 |
| **9** | control | 0 | 3,56 | **16,81** | 6,6 | 1 | 7,34 |
| **10** | control | 1,22 | 6,43 | 14,28 | 3,54 | 3 | 6,39 |
| **11** | control | 0,18 | 0,77 | 15,1 | 9,47 | 1 | 4,96 |
| **12** | control | 0,91 | 1,12 | 14,46 | 6,50 | 0 | 5,23 |
| **13** | control | 0,19 | 0,84 | 15,1 | 6,93 | 0 | 6,32 |
| **14** | SRMA acute | 0,38 | 1,05 | 9,47 | 3,49 | 229 | 15,14 |
| **14** | SRMA remission | 0,46 | 0,23 | 10,14 | 5,17 | 4 | n/a |
| **15** | SRMA acute | 0,29 | 1,37 | 0,07 | 1,76 | 2404 | 21,77 |
| **15** | SRMA remission | 2,23 | 1,2 | 0,86 | 2,53 | 1 | 10,20 |
| **16** | SRMA acute | 0,87 | 7,23 | 0,05 | 2,65 | 9500 | 24,27 |
| **16** | SRMA remission | 0 | 10,48 | 10,1 | 4,33 | 0 | 9,72 |
| **17** | SRMA acute | 0,63 | 3,96 | 0,86 | 2,53 | 3650 | 20,18 |
| **17** | SRMA remission | 0 | 0,67 | 11,7 | 5,07 | 0 | 13,98 |
| **18** | SRMA acute | 1,26 | 3,32 | 9,0 | 4,36 | 505 | 10,10 |
| **19** | SRMA acute | 24,64 | 12,86 | 7,76 | 3,50 | 1476 | 19,10 |
| **20** | SRMA acute | 1,67 | 0,12 | 10,50* | 7,13* | 130 | 19,43 |
| **21** | SRMA acute | 0,08 | 0,81 | 12,03* | 6,88* | 422 | 21,31 |
| **22** | SRMA acute | 0,25 | 1,64 | 4,14* | **11,80*** | 448 | 17,76 |
| **23** | SRMA acute | 27,41 | 21,4 | 1,69 | 3,96 | 800 | 26,45 |
| **24** | SRMA acute | **309,51** | 1,98 | 9,55 | 2,83 | 120 | 26,64 |
| **25** | SRMA acute | 126,04 | 0,02 | 0,29 | 4,15 | n/a | 29,20 |
| **26** | SRMA acute | 4,11 | 3,54 | 2,86* | 4,18* | n/a | 30,10 |
| **27** | SRMA acute | 58,39 | 3,25 | 1,48 | 6,65 | n/a | 14,07 |
| **28** | SRMA acute | 49,59 | 22,18 | 2,57 | 4,76 | n/a | 29,29 |
| **29** | SRMA acute | 2,34 | 16 | 9,35 | 9,48 | 549 | 7,91 |
| **30** | SRMA acute | 29,96 | 52,2 | 6,3 | 7,38 | 4064 | 20,72 |
| **31** | SRMA acute | 116,85 | **55,71** | 3,83 | 4,21 | 6288 | 33,43 |
| **32** | SRMA acute | 8,28 | 9,71 | 4,68 | 5,18 | 128 | 12,00 |
| **33** | SRMA acute | 0,72 | 39,51 | 8,81 | 8,94 | 824 | 13,31 |
| **34** | SRMA acute | 8,39 | 11,6 | 12,30 | 7,29 | 4128 | 22,00 |
| **35** | SRMA acute | 0 | 56,75 | 10,20 | 5,45 | 498 | 25,95 |
| **36** | SRMA acute | 58,29 | 2,24 | 1,26 | 8,26 | 7168 | 27,30 |
| **37** | SRMA acute | 51,94 | 9,6 | 0,15 | 6,20 | 17900 | 25,10 |
| **38** | SRMA acute | 58,84 | 15,19 | 6,97 | 7,51 | 3250 | n/a |
| **39** | SRMA acute | 13,84 | 3,64 | 4,92 | 3,26 | 230 | n/a |
| **40** | SRMA acute | 0,19 | 17,56 | 13,89 | 6,32 | 74 | 29,30 |
| **41** | SRMA acute | 58,84 | 2,24 | 6,14 | 4,46 | 225 | 14,40 |
| **42** | SRMA acute | 162,44 | 9,4 | 5,82 | 4,35 | 677 | n/a |
| **43** | SRMA acute | 162,64 | 28,59 | 3,83 | 5,17 | 2997 | n/a |
| **44** | SRMA acute | n/m | n/m | 5,65 | 5,21 | 1968 | 27,40 |
| **45** | SRMA acute | 43,23 | 5,2 | 3,5* | 5,53* | 1835 | 27,70 |
| **46** | SRMA acute | 9,7 | 9,7 | 10,58 | 5,98 | 29 | 16,90 |
| **47** | SRMA acute | 54,87 | 0,85 | 1,44 | 3,72 | 939 | n/a |

CSF: cerebrospinal fluid, ID: patient identification, µL: microliter, mL: milliliter, min: minute, n/a: not applicable, ng: nanogram, n/m: not measurable, pmol: picomol, SRMA: steroid-responsive meningitis-arteritis, WBC: white blood cell count

Outliers were marked with bold values and were identified using Grubbs´s testing.

WBC: 6 x 10^3^/µL to 12 x 10^3^/µL, leukocytes CSF: <5 leukocytes/3 µL CSF
